# Heterogeneity of glucose metabolism and uptake identifies distinct cancer cell and cancer stem cell phenotypes

**DOI:** 10.1186/s11658-025-00837-0

**Published:** 2026-01-17

**Authors:** Zuzana Tylichova, Martin Krkoska, Vaclav Hrabal, Michaela Stenckova, Borivoj Vojtesek, Philip J. Coates

**Affiliations:** RECAMO, Masaryk Memorial Cancer Institute, Zluty Kopec 7, Brno, 656 53 Czech Republic

**Keywords:** Glucose metabolism, Cancer stem cells, Mitochondria, ALDH, LDH, SDH, GLUT1

## Abstract

**Background:**

Tumor cells show phenotypic heterogeneity, including a small subpopulation of cancer stem-like cells (CSCs) that are responsible for maintaining tumor growth and metastasis. Altered glucose metabolism is a characteristic feature of cancer cells, which often display increased aerobic glycolysis alongside mitochondrial oxidative respiration (the Warburg effect). However, there is evidence that CSCs exhibit distinct glucose metabolism compared with the tumor cell bulk, with increased mitochondrial activity and oxidative respiration. Thus, identifying individual cells with different modes of glucose metabolism may serve as a common identifier of CSCs, and these metabolic differences would allow selective therapeutic targeting.

**Methods:**

We investigated the levels of enzymes involved in glycolysis and oxidative respiration, together with glucose uptake and mitochondrial membrane potential in individual cancer cells. These parameters were correlated with each other and with CSC markers.

**Results:**

We show considerable heterogeneity of metabolic markers in individual tumor cells. Surprisingly, high glucose uptake correlates with high mitochondrial membrane potential, indicating that increased oxidative respiration and aerobic glycolysis coexist rather than showing an inverse correlation. We also show that colonies derived from cells with high mitochondrial membrane potential exhibit heterogeneous metabolic parameters, demonstrating that metabolic profiles are not hard-wired. Public gene expression profiling data indicated similar inconsistent metabolic features of CSCs.

**Conclusions:**

The data reveal inherent heterogeneity and plasticity of glucose metabolism and mitochondrial membrane potential in tumor cells, with evidence for a subpopulation that possesses both increased glucose uptake and increased mitochondrial membrane potential, with implications for therapeutic targeting of metabolism in cancer.

**Graphical abstract:**

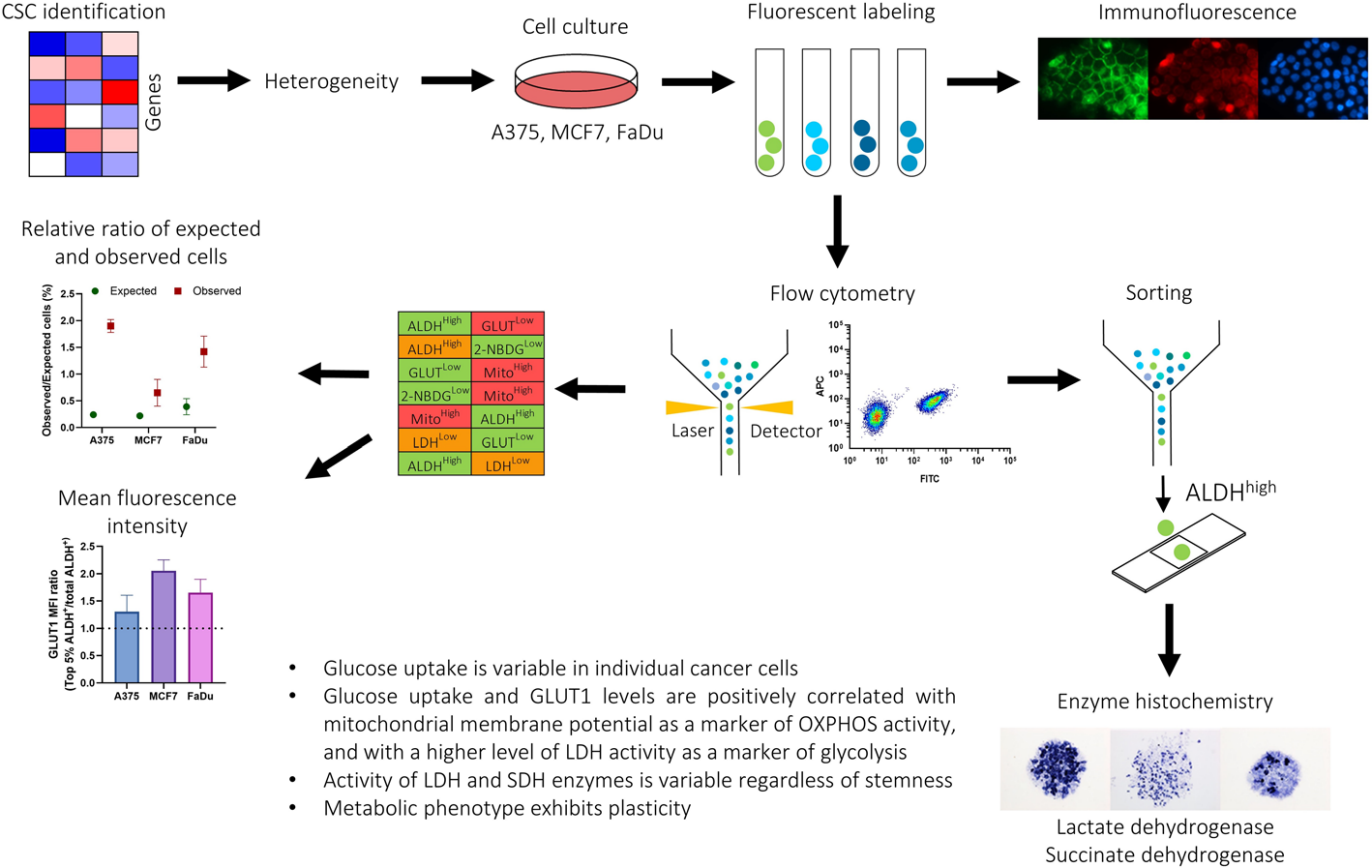

**Supplementary Information:**

The online version contains supplementary material available at 10.1186/s11658-025-00837-0.

## Background

Tumors are composed of mixed populations of non-malignant stromal cells and malignant cells that exhibit varying degrees of differentiation, including a population of slow cycling stem-like cells usually referred to as tumor-initiating cells or cancer stem-like cells (CSCs) [1, 2]. CSCs are defined as the unique population of tumor cells that possess self-renewing capacity through asymmetric cell division and undergo limited differentiation to produce the range of cell types present in the tumor. Similar to stem cells in normal tissues, CSCs represent a small fraction of the tumor [3, 4]. CSCs are identified mainly by specific cell surface proteins (e.g., CD24^−^/CD44^+^, Lgr5^+^, or CD133^+^), by high aldehyde dehydrogenase (ALDH) activity, or by their ability to efflux dyes to produce a side population [5]. However, rather than identifying the same cell population in a tumor, these markers identify different CSC subtypes that co-exist [6–8] and are spatiotemporally regulated, where cells may transition from one type to another [2, 8–11]. Functional tests for CSCs include the ability to form holoclones as adherent cells, to form spheroids in suspension culture, or the efficiency with which they form tumor xenografts in immunodeficient mice [4, 5, 12, 13].

The lack of consistency of individual markers in CSCs across different tumor types and CSC-subtypes creates a requirement to identify broad and reliable CSC characteristics. Such general features would allow therapeutic interventions that target multiple CSC-subtypes simultaneously. One relevant area is metabolism, where normal adult somatic stem cells show different metabolic properties from their differentiated progeny [14], although whether CSCs show similar alterations from the tumor bulk is unclear. Cancer cells commonly exhibit enhanced glycolysis even in the presence of sufficient oxygen, while still maintaining active mitochondrial oxidative phosphorylation (OXPHOS)—a phenomenon known as the Warburg effect [15–17]. As a consequence, cancer cells require high levels of glucose uptake, and often upregulate glucose transporters such as GLUT1 (encoded by *SLC2A1*) to facilitate their high glucose requirements [18, 19]. Indeed, increased glucose uptake is the basis of ^18^F-fluorodeoxyglucose positron emission tomography (FDG PET), the most common clinical cancer imaging modality [20]. As a consequence of Warburg metabolism, tumor cells show high levels of lactate dehydrogenase (LDH; associated with glycolysis) and pyruvate dehydrogenase kinases (PDK1/PDK4) that inhibit the tricarboxylic acid (TCA) cycle to downregulate aerobic respiration [21, 22]. Tumor cells also contain high levels of the bidirectional lactate transporter MCT1 (encoded by *SLC16A1*), which facilitates the excretion of excess lactate produced through glycolysis [16], while MCT4, commonly expressed in many cancers, functions almost exclusively as a lactate exporter [23].

These features of altered cancer cell metabolism refer to the tumor overall, and there is growing evidence that CSCs may show differences in glycolytic pathways, glucose uptake, lactate production, and mitochondrial oxidative respiration compared with their progeny [24–29]. These metabolic differences may therefore represent a characteristic feature of CSCs and could be used for therapeutic targeting of these cells, such as metformin that inhibits OXPHOS [30–34] or glucose analogs such as 2-deoxy-d-glucose that disrupt glycolysis [35–37]. Here, we performed a multi-modal investigation of metabolic enzymes, glucose transport, and mitochondrial mass and activity in individual cells of human cancer cell lines of diverse origins to investigate whether consistent differences can be identified that help to define a common CSC metabolic phenotype.

## Methods

### Cell lines

We used three human cancer cell lines that represent diverse common human malignancies; A375 (melanoma), FaDu (squamous cell carcinoma of head and neck), and MCF7 (estrogen receptor positive luminal breast cancer), all obtained from the American Type Culture Collection (Manassas, VA, USA) and authenticated by STR DNA profiling (Eurofins Genomics, Ebersberg, Germany). Unless stated otherwise, cells were cultured in high glucose Dulbecco’s modified Eagle medium (DMEM; D5648 Sigma Aldrich, St Louis, MO, USA) supplemented with 100 U/ml penicillin/100 µg/ml streptomycin (Biosera XC-A4122/100; BioTech a.s. Prague, Czech Republic), 1% sodium pyruvate, and 10% heat-inactivated fetal bovine serum (Biosera S-FBS-SA-015). Cells were maintained at 37 °C, 5% CO_2_ and 95% humidity, and passaged by trypsinization when they reached approximately 80% confluency. For experimental use, cells were counted using a TC20 automated cell counter (BioRad spol s.r.o., Prague, Czech Republic), and were seeded at appropriate density to reach 80% confluency at the time of analysis of the particular experiment.

### Flow cytometry

Cells measured by flow cytometry were gated on the basis of forward and side scatter (FSC versus SSC) to exclude debris and doublets and select the main cell population. A total of 10,000 events were acquired per sample. Cell viability was assessed using a live/dead assay (Invitrogen L34955), and only viable cells were included in analyses. Samples were measured on FACS Aria III or FACS Verse (BD Biosciences, San Jose, CA, USA). Data were analyzed using BD FACSDiva™ 7.0 and BD FACSuite v1.0.6 software.

### ALDH activity

Cells were trypsinized, rinsed, and centrifuged. Cell pellets (5 × 10^5^ cells per sample) were resuspended in buffer with verapamil and AldeRed or AldeGreen (Sigma-Aldrich, SCR150 and SCR151, respectively). The ALDH inhibitor diethylaminobenzaldehyde (DEAB) was added to replicate cells as a negative control. Cells were incubated for 30 min at 37 °C, centrifuged at 300 *g* for 5 min and analyzed by flow cytometry.

### Glucose uptake

In initial experiments, cells in suspension were incubated with different concentrations of the fluorescent glucose analog 2-(*N*-(7-nitrobenz-2-oxa-1,3-diazol-4-yl)amino)-2-deoxyglucose (2-NBDG; Thermo Fisher Scientific, Waltham MA, USA; N13195) in low glucose (1 g/l) or no glucose DMEM but containing the same concentrations of glutamine (Thermo Fisher Scientific; Gibco 11880-028 or 11966025) for varying periods of time prior to flow cytometry. In further experiments, standard DMEM (4.5 g/l) was replaced by low glucose DMEM (1 g/l) for 6 h before treatment with 60 µM 2-NBDG for 30 min. After washing with phosphate-buffered saline (PBS), cells were trypsinized and 2-NBDG fluorescence analyzed by flow cytometry.

### GLUT1 analyses

For flow cytometry, cells (5 × 10^5^ per sample) were fixed in 4% formaldehyde for 10 min at room temperature, washed in PBS and stained with AF647-conjugated GLUT1 antibody (195020 Abcam, Cambridge, UK), diluted 1:500, or unconjugated GLUT1 antibody (1:500, Abcam 15309) for 1 h at room temperature. Non-conjugated primary antibody was detected with AF488-conjugated goat anti-rabbit (1:500, Thermo Fisher Scientific, Invitrogen, A32731) for 1 h at room temperature. Appropriate isotype controls for AF647 (1:1000, Cell Signaling Technology, Danvers, MA, USA, 2985) and secondary antibody in the absence of primary antibody were used as negative controls.

### Mitochondrial membrane potential

Cells were treated with 100 nM MitoTracker Deep Red FM (M22426, Thermo Fisher Scientific) or 100 nM MitoMark Red (6445, Tocris Bioscience, Bristol, UK) for 30 min at 37 °C, trypsinized, rinsed, and analyzed by flow cytometry. To assess specificity, cells were treated with 10 μM valinomycin (Invitrogen V1644) to dissipate mitochondrial membrane potential, resulting in reduced fluorescence intensity.

### Cell cycle

For cell cycle analysis, cells (5 × 10^5^ per sample) were fixed in 70% EtOH for 30 min at 4 °C and stained with Vindel solution (1 M Tris pH 8, RNAse A 100 µg/ml, 10% Triton, propidium iodide 50 µg/ml, and distilled water) for 30 min at room temperature. To combine cell cycle with another parameter, cells were stained with AF647-conjugated GLUT1 antibody (Abcam, 195020) or MitoMark Red (Tocris, 6445), as described above, prior to fixation with EtOH. Samples were measured by the FACS Verse flow cytometer. Cell cycle distribution was analyzed using ModFit LT 6.0 software.

### Flow cytometry data evaluation

Cells for flow cytometry analyses of two different parameters in the same experiment were selected as the 5% of cells showing the specific characteristics proposed for CSCs. These cells were then analyzed for the other phenotype examined in the experiment to investigate whether enrichment for one trait also enriched for cells with the other trait (e.g., the top 5% of Mito^+^ versus the top 5% of ALDH^+^). The use of a 5% cutoff for each parameter was derived from observations that CSCs are a small subpopulation of cells, representing between approximately 2% and 10% of the total cancer cell population [38–40], and the selection of 5% as a threshold for CSC enrichment has been used previously to good effect [6]. With this strategy we defined two populations, one representing the putative CSCs (5%), and the second population representing cells that are not CSCs (95%). The average level of the parameter is not important here, given that the “average” cell is not a CSC. Using this approach for statistical evaluation, 0.25% of the cells are expected to be present in the double gated region (5% × 5%) if the two parameters are unrelated, representing the null hypothesis. However, the 5% gates for each parameter are drawn manually for each replicate in each experimental setup, and the actual percentage of cells expected to be included in the analyzed gate is slightly variable. The “expected” values assuming the null hypothesis are then compared with the values “observed” in the experimental results, using statistical tests to investigate deviation from the null hypothesis.

To assess enrichment of selected markers within the 5% population, mean fluorescence intensity (MFI) values for both groups were compared. Bar graphs show the relative level of parameter A (measured as MFI) in the top 5% of parameter B^+^ cells compared with the entire population. Ratios from three independent experiments were analyzed using a one-sample *t*-test, comparing the mean of each group with the theoretical value of 1 (no difference between populations).

### Cell sorting

Cells were stained for mitochondrial membrane potential (MitoTracker Deep Red) or ALDH activity (AldeRed) as described above. Cells with the highest mitochondrial membrane potential (top 5%) or the highest ALDH activity (top 5%) were sorted using a FACS Aria onto coverslips in 12-well plates and allowed to attach and form colonies. LDH and SDH enzyme histochemistry was performed as described above.

### Colony preparation, and LDH and SDH enzyme histochemistry

Cells were seeded at cloning density onto sterile 22 mm × 22 mm coverslips in 6-well plates and allowed to form colonies for 7–10 days. After rinsing with PBS, cells were fixed with 4% formaldehyde for 5 min at room temperature, washed three times in PBS and incubated for 15 min at 37 °C in assay buffer adapted from [41], using reagents from Sigma-Aldrich. For lactate dehydrogenase (LDH): 50 mM Tris pH 7.4, 5 mM sodium azide (S2002), 0.3 mM methoxyphenanzine methosulfate (M8640), 3 mM NAD^+^ (N1511), and 100 mM sodium lactate (71718). For succinate dehydrogenase (SDH): 50 mM Tris pH 8.0, 5 mM sodium azide (S2002), 0.2 mM phenanzine methosulfate (P9625), and 50 mM sodium succinate (14160). All assay reagents were prepared freshly and contained 600 μM nitroblue tetrazolium chloride (NBT; 1.24823). Negative control reactions in the absence of enzyme substrate were performed for each enzyme. After 10–30 min, depending on the enzyme, cells were rinsed repeatedly in distilled water, allowed to dry, and mounted on glass slides with Mowiol (Sigma-Aldrich, Millipore 475904) for light microscopy. Specimens were examined using a Nikon Eclipse Ci-L with a 10× objective. Cells were analyzed using ImageJ.

For combinations of enzyme histochemistry with mitochondrial mass, cells were treated with 50 nM MitoMark Green (Tocris, 6444) for 30 min at 37 °C and then fixed with 4% formaldehyde for 5 min. NBT was replaced with the fluorescent substrate 5 mM 5-cyano-2,3-di-(p-tolyl) tetrazolium chloride (Sigma Aldrich, 94498). ProLong™ Glass Antifade Mountant with NucBlue™ Stain (Thermo Fisher P36981) was used for nuclei staining and slide mounting. Specimens were examined using an EVOS FL Cell Imaging System (Thermo Fisher) with a 40× objective.

### Immunofluorescence combined with mitochondrial membrane potential

Cells were seeded at cloning density onto 15 mm × 15 mm coverslips in 12-well plates and allowed to attach and form colonies. For mitochondrial membrane potential or mitochondrial mass analysis, cells were treated with 100 nM MitoMark Red or 100 nM MitoMark Green for 30 min at 37 °C. Cells were rinsed and fixed in 4% formaldehyde for 10 min, washed in PBS, and permeabilized in 0.1% Triton-X for 5 min for cytoplasmic antigen staining. Cells were incubated with blocking buffer (1% bovine serum albumin [BSA] in PBS) and stained with primary antibodies diluted in blocking buffer: AF488-conjugated ALDH1A1 (1:100; Abcam, 195254), AF647-conjugated ALDH1A1 (1:100; Abcam, 195255), AF647-conjugated GLUT1 (1:100; Abcam, 195020), or non-conjugated GLUT1 (1:100; Abcam, 15309) overnight at 4 °C. Non-conjugated primary antibody was detected with AF488-conjugated goat anti-rabbit (1:500; Invitrogen, A32731) for 1 h at room temperature. Appropriate isotype controls (1:100; Cell Signaling, 2985 and 2975) or secondary antibody in the absence of primary antibody were used as negative controls. Antifade Mountant with NucBlue™ Stain (Thermo Fisher, P36981) was used for nuclei staining and slide mounting. Specimens were examined using an EVOS FL Cell Imaging System (Thermo Fisher) with 40× objective. Cells were analyzed using QuPath 0.6.0 software.

### Immunofluorescence quantification

Immunofluorescence images of three meroclone and three holoclone colonies (for each marker pair) were analyzed in QuPath 0.6.0. Colonies were manually annotated, and cells were detected using a cell detection algorithm in QuPath. Single-cell mean fluorescence intensity values were extracted for each cell for their respective channels. Data were exported as text files and subsequently analyzed in GraphPad Prism 8.4.3 and MS Excel. Pairwise associations between markers were assessed using Spearman’s rank correlation on pooled single-cell data and analyzed separately for holoclone and meroclone colonies. Correlation coefficients are reported in graphs as Spearman’s *ρ* together with associated *p*-values. For analysis of cells with the top 5% fluorescence intensity, single cell mean fluorescence intensities were normalized within each colony and channel by dividing cell intensity by the median fluorescence intensity of all cells in that colony. Normalized values were used for the analysis. For each marker, cells belonging to the top 5% fluorescence intensity distributions within a colony were identified. The mean ratios of such high-intensity cells were then calculated for holoclones compared with meroclones in MCF7 and FaDu using two-tailed Student’s *t*-tests.

### Bioinformatics analyses of metabolic parameters in CSC-enriched populations

Gene expression profiles of CSC and non-CSC populations purified using individual CSC markers or functional assays were retrieved from the Gene Expression Omnibus (https://www.ncbi.nlm.nih.gov/geo/) and annotated in GEO2R as either CSC or non-CSC populations according to the author’s experimental protocol. The mRNA levels of genes that regulate glycolysis and glucose import/lactate export (*LDHA*, *HK1*, *HK2*, *PFKB3*, *IDH1*, *PDK1*, *PDK4*, *SLC2A1*, and *SLC16A1*), TCA cycle and mitochondrial activity (*SDHA*, *SDHAF1*, *IDH2*, *IDH3A*, *NDUFA5*, *CYC1*, *TFAM*, *TOMM40*, *VDAC1*, and *PPARGC1A*), pentose phosphate pathway (*G6PD* and *TALDO1*), and fatty acid/lipid metabolism (*ACLY*, *ACACA*, *FASN*) along with five commonly used CSC marker genes (*ALDHA1/2*, *CD44*, *CD133*, *LGR5*, and *CD24*) were retrieved using the profile graph and sample values tools. As recommended by GEO, on the basis of manual examination of boxplots and density plots, some datasets required normalization before analysis. The specific datasets analyzed are provided in the Results section.

### Statistics

Experimental results are provided as the mean ± SEM of three independent experiments. One-way ANOVAs followed by Tukey’s tests or nonparametric Mann–Whitney tests were used for statistical analyses. For GEO data, results are shown as log_2_ fold change in the CSC population compared with the non-CSC population, and *p*-values were calculated using two-way unpaired *t*-tests assuming unequal variance. Paired *t*-tests were used with data derived from paired samples (for example, patient samples isolated as CSC and non-CSC populations from the same tumor). Statistical significance was determined as *p < 0.05, **p < 0.01, and ***p < 0.001.

## Results

### CSC markers identify different cell populations

**Fig. 1:**
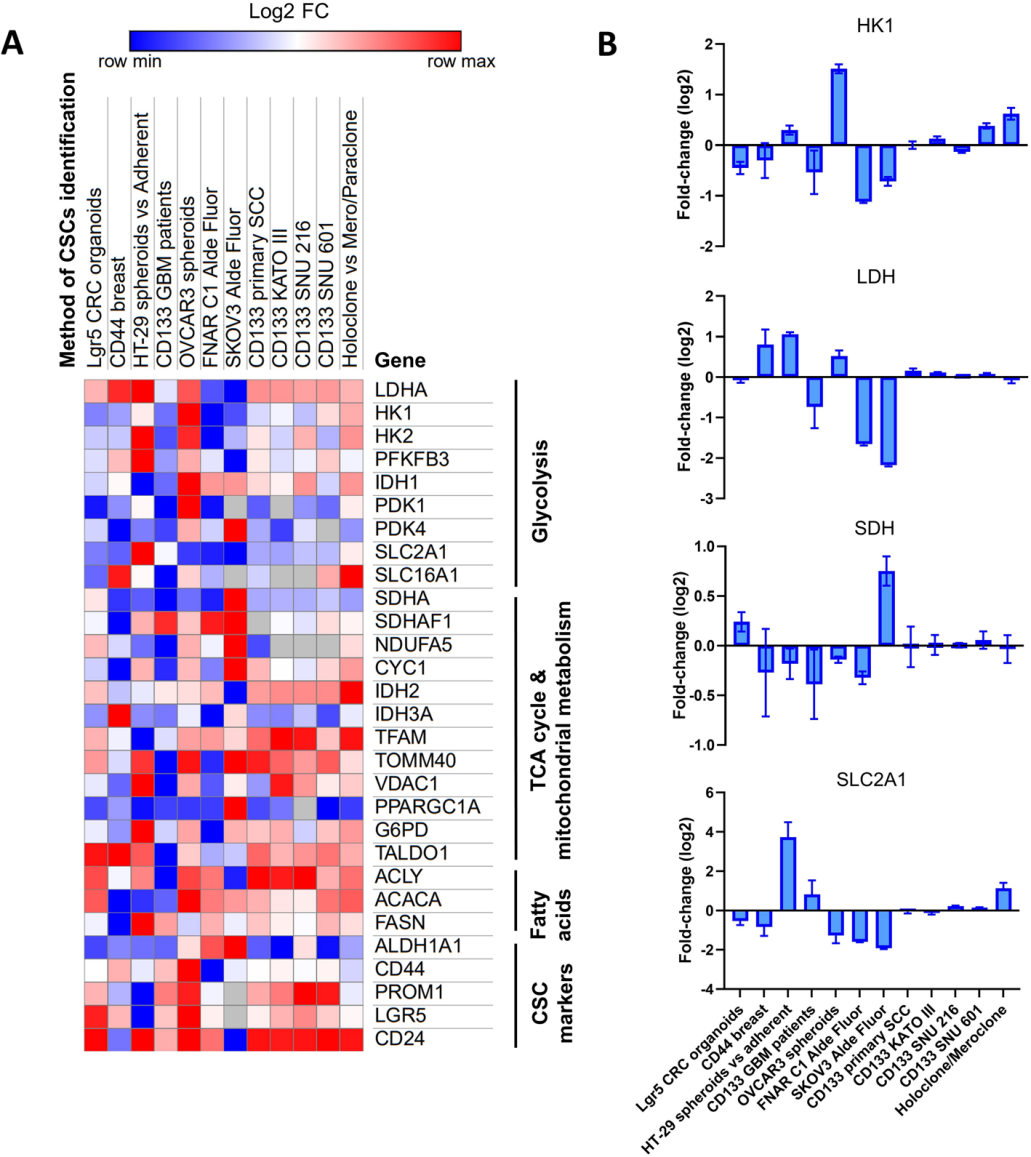
CSC markers identify different cell population. **a** Heatmap shows the expression of selected genes associated with glycolysis, the TCA cycle and mitochondrial metabolism, Fatty acids and CSC markers across 12 different CSC identification methods. The heatmap was generated using the online tool Morpheus (https://software.broadinstitute.org/morpheus/), colors reflect relative gene expression (log_2_ fold change), where blue denotes downregulation and red denotes upregulation. **b** Four genes were selected (*HK1*, *LDH*, *SDH*, and *SLC2A1*) for detailed comparison and are shown separately in bar graphs, highlighting key differences between identification approaches. Data are presented as mean log_2_ fold change. Error bars in bar plots indicate standard error of the mean (SEM)

Public gene expression profiling data were used to investigate glycolytic and TCA cycle enzymes, pentose phosphate pathway enzymes, mitochondrial biogenesis, and glucose import/lactate exporters in cancer cells isolated using various CSC markers. These analyses revealed inconsistencies between commonly used CSC markers, where individual markers were not co-regulated in the CSC populations identified in each experimental setup. These findings are in keeping with previous observations that individual CSC markers identify different tumor cells rather than identifying a single, common population of CSCs [6–8]. All of the datasets examined showed statistically significant differences in individual metabolic parameters in the isolated CSC population compared with the non-CSC population (Fig. 1a), agreeing with the heterogeneity of metabolism that we identified using protein levels and functional assays presented in this study (see below). However, changes of mRNAs involved in the same process were not consistent, either within a dataset or between different datasets. Thus, some experiments showed increased mRNAs coding for enzymes involved in glycolysis and reduced TCA enzymes in the putative CSC populations, whereas other datasets showed no difference or the opposite trend (Fig. 1). Moreover, the same criterion used for CSC identification in different cell lines in the same laboratory or on the same cell line in different laboratories did not identify the same metabolic profiles. Importantly, the isolated stem cell population sometimes showed inverse changes in different mRNAs involved in the same biological process (Fig. 1b). However, the interpretation of cell populations in Fig. 1, based on a limited set of genes with modest fold changes, should be considered with caution, and extrapolating gene expression profiles to metabolic properties carries inherent limitations, particularly that mRNA level does not always correlate with protein or activity level [42], as many metabolic and mitochondrial enzymes are regulated by post-transcriptional and post-translational mechanisms [43, 44]. While substantial work has explored links between transcriptomic and metabolic states, only a small number of studies have directly correlated RNA sequencing data with experimentally measured metabolic activity [45], and we view our approach as an initial step toward integrating these complementary datasets.

### Glucose uptake is variable in individual cancer cells

In this study, we included cell lines A375, MCF-7, and FaDu as models representative of prevalent and widely studied human cancers of diverse origin—melanoma, luminal breast adenocarcinoma, and head-and-neck squamous cell carcinoma, respectively. All experiments were performed under identical, asynchronous conditions (all cell lines were measured at the same time post-passage, without any external treatment or synchronization). To ensure that the cells maintained standard characteristics and viability, we evaluated both cell viability (Supplementary Fig. S1A) and cell cycle profiles (Supplementary Fig. S1B).

**Fig. 2:**
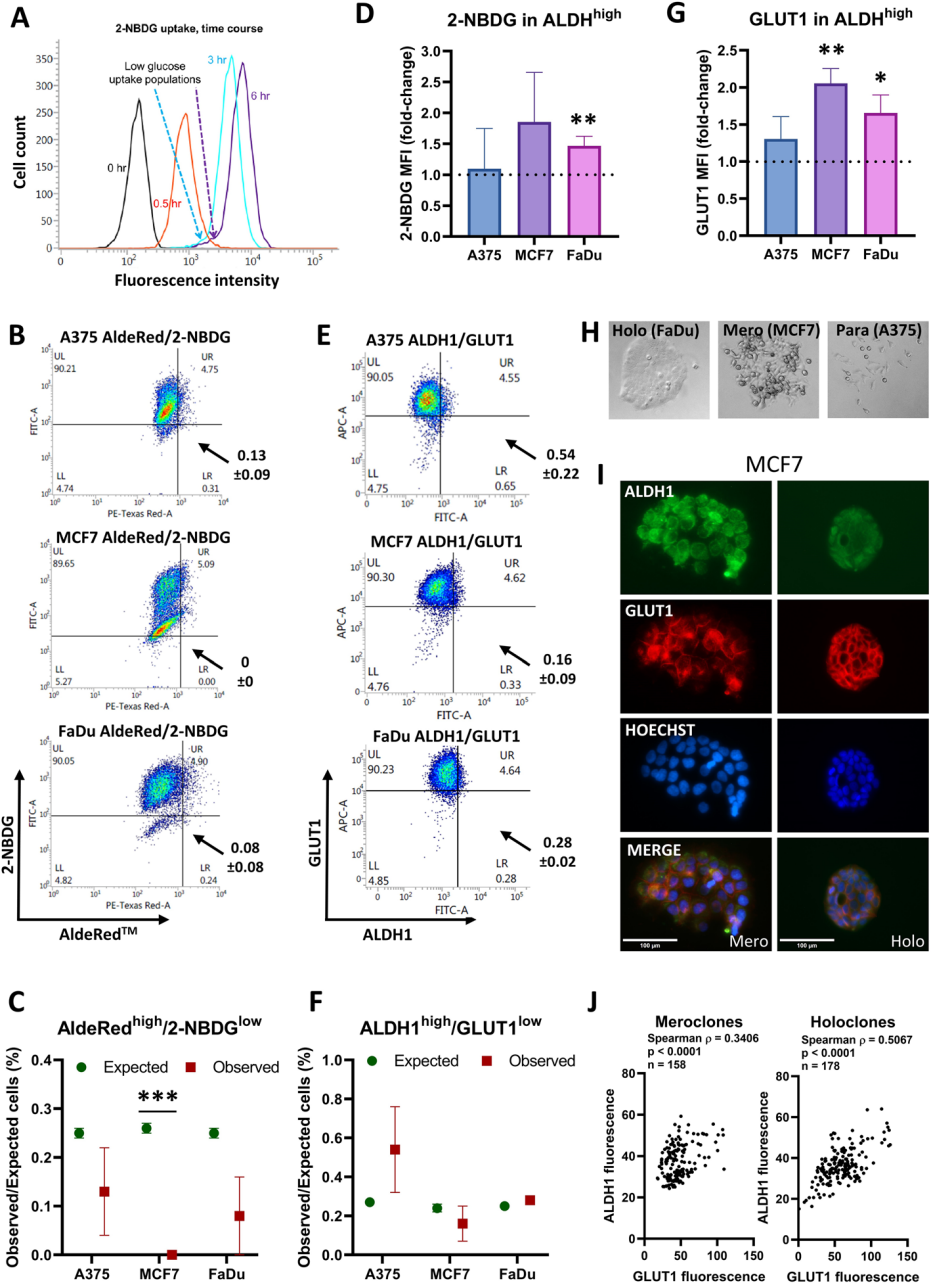
Glucose uptake is variable in individual cancer cells. **a** Flow cytometry detection of glucose uptake (2-NBDG) in MCF7 cells at the indicated time points. The dotted arrows point to a population of cells with low fluorescence at 3 and 6 h. The histogram represents a preliminary experiment used to optimize the measurement conditions. **b** Glucose uptake (2-NBDG) and ALDH activity (AldeRed) in A375, MCF7, and FaDu cells. Uptake of 2-NBDG was measured at 30 min. The quadrants identify cells in the top 5% of AldeRed activity and the lowest 5% of 2-NBDG uptake. The values shown next to the graphs represent the mean ± SD from three independent experiments, whereas the numbers displayed in the quadrants correspond to the representative density plot shown in the figure. **c** The expected percentage of cells in the region of interest if there is no relationship between the variables, compared with the observed percentage in that region (mean ± SEM, *n* = 3 biological replicates). ***p < 0.001. **d** Bar graph showing the relative uptake of 2-NBDG (measured as MFI) in the top 5% of ALDH^+^ cells compared with the entire ALDH^+^ population across three cell lines. Values are normalized to the mean MFI of 2-NBDG in the whole ALDH^+^ population for each respective cell line. The dashed horizontal line at 1.0 indicates no difference. Significant difference **p < 0.01. **e** Glucose transport (GLUT1) and ALDH1 activity in A375, MCF7, and FaDu cells. The quadrants identify cells in the top 5% of ALDH1 activity and the lowest 5% of GLUT1 transport. The values shown next to the graphs represent the mean ± SD from three independent experiments, whereas the numbers displayed in the quadrants correspond to the representative density plot shown in the figure. **f** The graph shows the expected percentage of cells in the region of interest if there is no relationship between the variables, compared with the observed percentage in that region (mean ± SEM, *n* = 3 biological replicates). **g** The relative expression of GLUT1 (measured as MFI) in the top 5% of ALDH^+^ cells compared with the entire ALDH^+^ population across three cell lines. Values are normalized to the mean MFI of GLUT1 in the whole ALDH^+^ population for each respective cell line. The dashed horizontal line at 1.0 indicates no difference. Significant difference *p < 0.05, **p < 0.01. **h** Representative images of clones with different morphology. **i** Representative images of MCF7 colonies stained with ALDH1 (green), GLUT1 (red), and Hoechst (blue). The scale bar represents 100 μm. Holo/mero/para clone next to the scale bar denotes the colony morphology type. **j** Fluorescence quantification of holoclones and meroclones using QuPath. Pairwise associations between markers were assessed using Spearman’s rank correlation. Correlation coefficients are reported in graphs as Spearman’s *ρ* together with associated *p*-values. The number of analyzed cells is indicated in the graphs as *n*

To asses glucose uptake by individual tumor cells, we used flow cytometry of cells exposed to the fluorescent glucose analog 2-NBDG, which is actively transported into cells in a concentration- and time-dependent manner [46]. In initial experiments, 2-NBDG accumulation was quenched by increasing the glucose concentration in the culture medium—undetectable accumulation in conventional DMEM (4.5 g/l glucose), increasing in low glucose DMEM (1 g/l glucose), and further increased in DMEM in the absence of glucose, indicating the specificity of the assay for glucose uptake. To avoid potential complications of complete glucose depletion and cell death during culture, cells were incubated in low glucose medium for 6 h prior to treatment with 60 µM 2-NBDG in the same medium, and collected 0, 0.5, 3, and 6 h later. As shown in Fig. 2a, there are subpopulations of MCF7 cells at 3 and 6 h that have taken up less 2-NBDG than the bulk of the cells, indicated by the dotted arrows (blue at 3 h and purple at 6 h). From this time course, all subsequent experiments used low glucose DMEM (1 g/l) for 6 h before treatment with 60 µM 2-NBDG for 30 min. Similar variabilities in 2-NBDG uptake were seen for MCF7, FaDu, and A375 cells (Fig. 2b).

We combined 2-NBDG with AldeRed to investigate the relationship between glucose uptake and ALDH activity as a commonly used CSC marker. There was marked heterogeneity of 2-NBDG uptake in individual cells, varying by approximately 50- to 100-fold in different cells in each cell line, with a distinct population of cells showing low uptake, most noticeably in MCF7 and FaDu, but also present in A375 (Fig. 2b). To identify the AldeRed^high^ CSC population, we gated the 5% of cells showing the highest AldeRed fluorescence, and analyzed these cells for their 2-NBDG uptake levels. On the basis that CSCs may not rely on Warburg metabolism, we gated the 5% of cells with the lowest 2-NBDG fluorescence for comparison with ALDH activity. According to the null hypothesis that the two parameters are not associated with each other, 0.25% (5% of 5%) of cells are expected to be present within the double gated population (denoted as “expected cell percentage” in Fig. 2c), which is compared to the percentage of cells observed experimentally (denoted as the “observed cell percentage”). In contrast to the notion that AldeRed^high^ cells should show lower glucose uptake than the majority of cells, 2-NBDG^low^ cells were not enriched in the AldeRed^high^ cell population of A375 or FaDu (*p* = 0.308 and *p* = 0.151, respectively), whereas the AldeRed^high^ population showed a statistically significant lack of 2-NBDG^low^ cells in MCF7 (*p* = 0.0007) (Fig. 2c; Supplementary Fig. S2). Indeed, ALDH^high^ cells showed a significantly different MFI of 2-NBDG in FaDu cells (*p* < 0.01), but not in A375 or MCF7 (Fig. 2d).

We also measured glucose transporter GLUT1 levels on the cell surface by flow cytometry as another indicator of glucose uptake. GLUT1 levels were also highly variable in individual cells in each cell line, with a more than 1000-fold difference between the highest and lowest levels in individual cells, and a clear tail of cells with low GLUT1 (Fig. 2e; Supplementary Fig. S3A, B). We were particularly interested to identify whether the AldeRed^high^ population of cells (containing the ALDH^+^ CSC population) correlated with lower levels of GLUT1 to identify cells that do not require high glucose uptake for Warburg anabolism. Similarly to 2-NBDG uptake, the ALDH^high^ population of tumor cells did not associate with low GLUT1 levels (A375, *p* = 0.325; MCF7, *p* = 0.481; FaDu, *p* = 0.236) (Fig. 2f; Supplementary Fig. S3B). GLUT1 MFI was elevated in both MCF7 (*p* < 0.01) and FaDu cells (*p* < 0.05), whereas A375 cells showed no significant change (Fig. 2g).

We also used dual immunofluorescence staining of ALDH1 protein and GLUT1 in cell-line-derived colonies to investigate the association with different clone morphologies, where holoclones contain tightly packed cells and represent stem cell-derived colonies, compared with less densely packed meroclones representing progenitor cells, and paraclones representing the most highly differentiated population (Fig. 2h). Membrane staining of GLUT1 was seen in MCF7 and FaDu cells, but not in A375 cells, which form loose colonies and did not show classical holoclone/meroclone morphologies (Fig. 2i; Supplementary Fig. S4A). GLUT1 levels varied between individual colonies, and staining was more pronounced in the center of holoclone-like colonies of MCF-7 (Fig. 2i) and FaDu cells (Supplementary Fig. S4A) similar to the predominant staining of CSC markers CD44 and CD133 in the central regions of holoclones seen before [38, 47]. In both colony types, a positive correlation was observed in individual MCF7 cells between GLUT1 and ALDH1 fluorescence (Spearman *ρ* = 0.3406, *p* < 0.0001 for meroclones and *ρ* = 0.5067, *p* < 0.0001 for holoclones) (Fig. 2j). Strong correlations were also found in FaDu cells (Spearman *ρ* = 0.8438, *p* < 0.0001 for meroclones and *ρ* = 0.8168, *p* < 0.0001 for holoclones) (Supplementary Fig. S4B).

These data highlight the heterogeneity of glucose metabolism in individual cancer cells, and clearly indicate that CSCs identified by ALDH activity or ALDH1 protein levels do not show reduced glucose uptake, but may instead show increases in these parameters in a cell-type specific manner.

### Mitochondrial membrane potential is not associated with reduced glucose uptake or GLUT1 levels

**Fig. 3:**
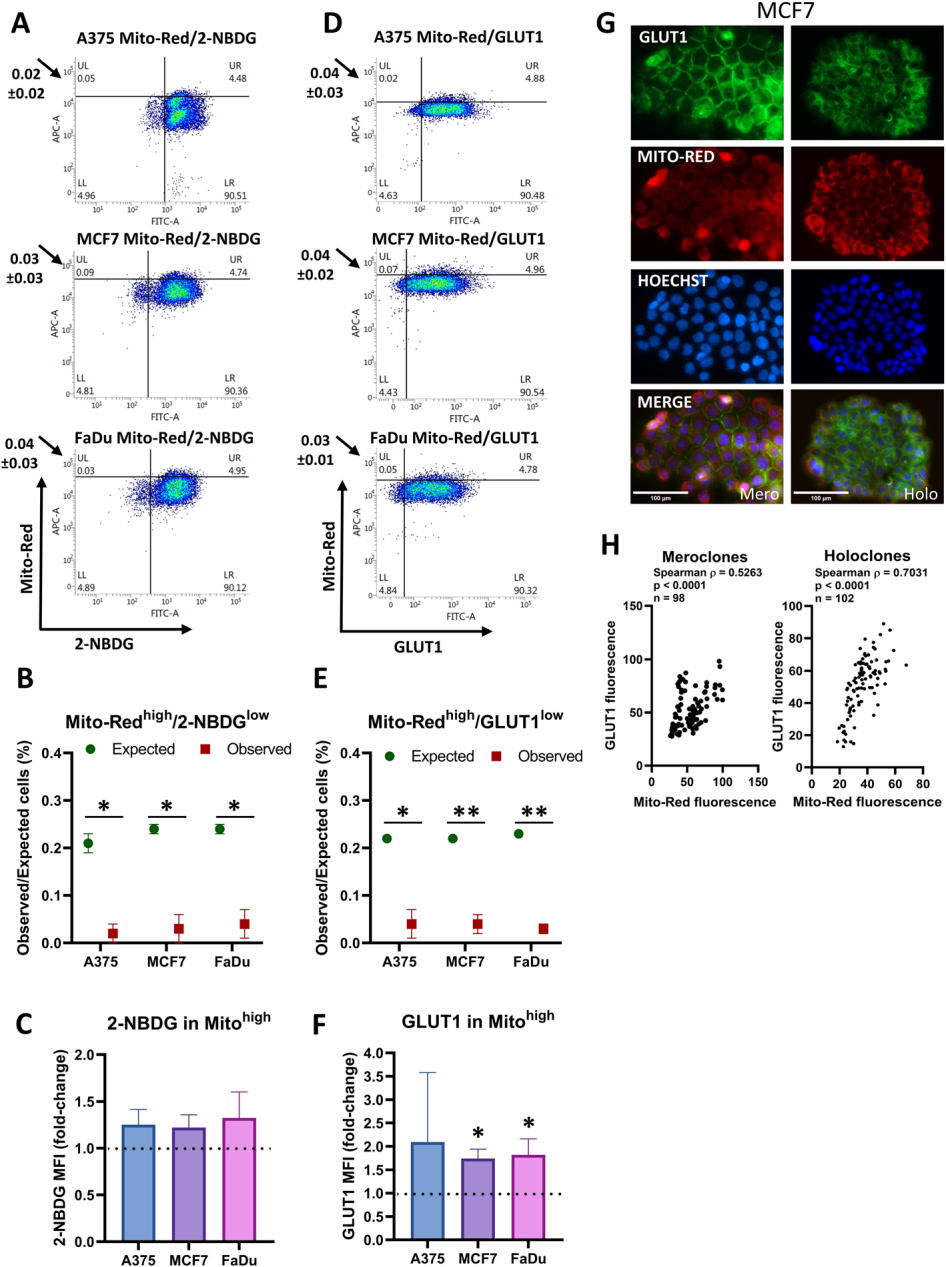
Mitochondrial membrane potential is not associated with reduced glucose uptake or GLUT1 levels. **a** Glucose uptake (2-NBDG) and mitochondrial membrane potential (Mito-Red) in A375, MCF7, and FaDu cells. Uptake of 2-NBDG was measured at 30 min. The quadrants identify cells in the top 5% of Mito-Red activity and the lowest 5% of 2-NBDG uptake. The values shown next to the graphs represent the mean ± SD from three independent experiments, whereas the numbers displayed in the quadrants correspond to the representative density plot shown in the figure. **b** The expected percentage of cells in the region of interest if there is no relationship between the variables, compared with the observed percentage in that region (mean ± SEM, *n* = 3 biological replicates). *p < 0.05. **c** The relative uptake of 2-NBDG (measured as MFI) in the top 5% of Mito-Red^+^ cells compared with the entire Mito-Red^+^ population across three cell lines. Values are normalized to the mean MFI of 2-NBDG in the whole Mito-Red^+^ population for each respective cell line. The dashed horizontal line at 1.0 indicates no difference. **d** Glucose transport (GLUT1) and mitochondrial membrane potential (Mito-Red) in A375, MCF7, and FaDu cells. The quadrants identify cells in the top 5% of Mito-Red activity and the lowest 5% of GLUT1 transport. The values shown next to the graphs represent the mean ± SD from three independent experiments, whereas the numbers displayed in the quadrants correspond to the representative density plot shown in the figure. **e** The expected percentage of cells in the region of interest if there is no relationship between the variables, compared with the observed percentage in that region (mean ± SEM, *n* = 3 biological replicates). *p < 0.05, **p < 0.01. **f** The relative levels of GLUT1 (measured as MFI) in the top 5% of Mito-Red^+^ cells compared with the entire Mito-Red^+^ population across three cell lines. Values are normalized to the mean MFI of GLUT1 in the whole Mito-Red^+^ population for each respective cell line. A dashed horizontal line at 1.0 indicates no difference. **p < 0.01. **g** Representative images of MCF7 colonies stained with GLUT1 (green), Mito-Red (red), and Hoechst (blue). The scale bar represents 100 μm. Holo/mero/para clone next to the scale bar denotes the colony morphology type. **h** Fluorescence quantification of holoclones and meroclones using QuPath. Pairwise associations between markers were assessed using Spearman’s rank correlation. Correlation coefficients are reported in graphs as Spearman’s *ρ* together with associated *p*-values. The number of analyzed cells is indicated in the graphs as *n*

Mitochondrial membrane potential of individual cells was measured using two probes dependent on mitochondrial membrane potential [48]. MitoTracker Deep Red (Thermo Fisher) was applied in flow cytometry experiments, owing to its far-red emission spectrum and minimal overlap with the green channel. In immunofluorescence experiments, we used MitoMark Red (Tocris), which was compatible with the respective panel of fluorophores and microscope settings. Both probes provided comparable results, confirming the robustness of our observations. To investigate the relationship of mitochondrial membrane potential with glucose uptake, we compared MitoMark-Red with 2-NBDG accumulation, and with GLUT1 levels. MitoMark-Red showed approximately fivefold variation in individual cells in each cell line under normal growth conditions (Fig. 3d), with increasing variability under the low glucose conditions used to measure 2-NBDG uptake (Fig. 3a). We were particularly interested to determine whether high mitochondrial membrane potential as a marker for cells that favor OXPHOS was associated with relatively low glucose transporter levels as a marker of cells with less reliance on Warburg metabolism. In contrast, the 5% of cells with the highest mitochondrial membrane potential was enriched with cells with higher 2-NBDG uptake (A375, *p* = 0.011; MCF7, *p* = 0.022; FaDu, *p* = 0.012) (Fig. 3b, c), and with high GLUT1 levels in all three cell lines (A375, *p* = 0.021; MCF7, *p* = 0.005; FaDu, *p* = 0.001) (Fig. 3d, e; Supplementary Figs. S5 and S6). The MFI of GLUT1 was elevated in all cell lines, which was not significant in A375 cells owing to the high variability, but was statistically significant in both MCF7 and FaDu cells (*p* < 0.05) (Fig. 3f).

We also evaluated differences in mitochondrial membrane potential in colonies. Mitochondrial membrane potential varied in individual cells within colonies (Fig. 3g; Supplementary Fig. S7). Positive correlations were observed between GLUT1 and Mito-Red fluorescence in MCF7 cells (Spearman *ρ* = 0.5263, *p* < 0.0001 for meroclones and *ρ* = 0.7031, *p* < 0.0001 for holoclones) (Fig. 3h). Similar values were observed in FaDu cells (Spearman *ρ* = 0.6333, *p* < 0.0001 for meroclones and *ρ* = 0.5956, *p* < 0.0001 for holoclones) (Supplementary Fig. S7B).

**Fig. 4:**
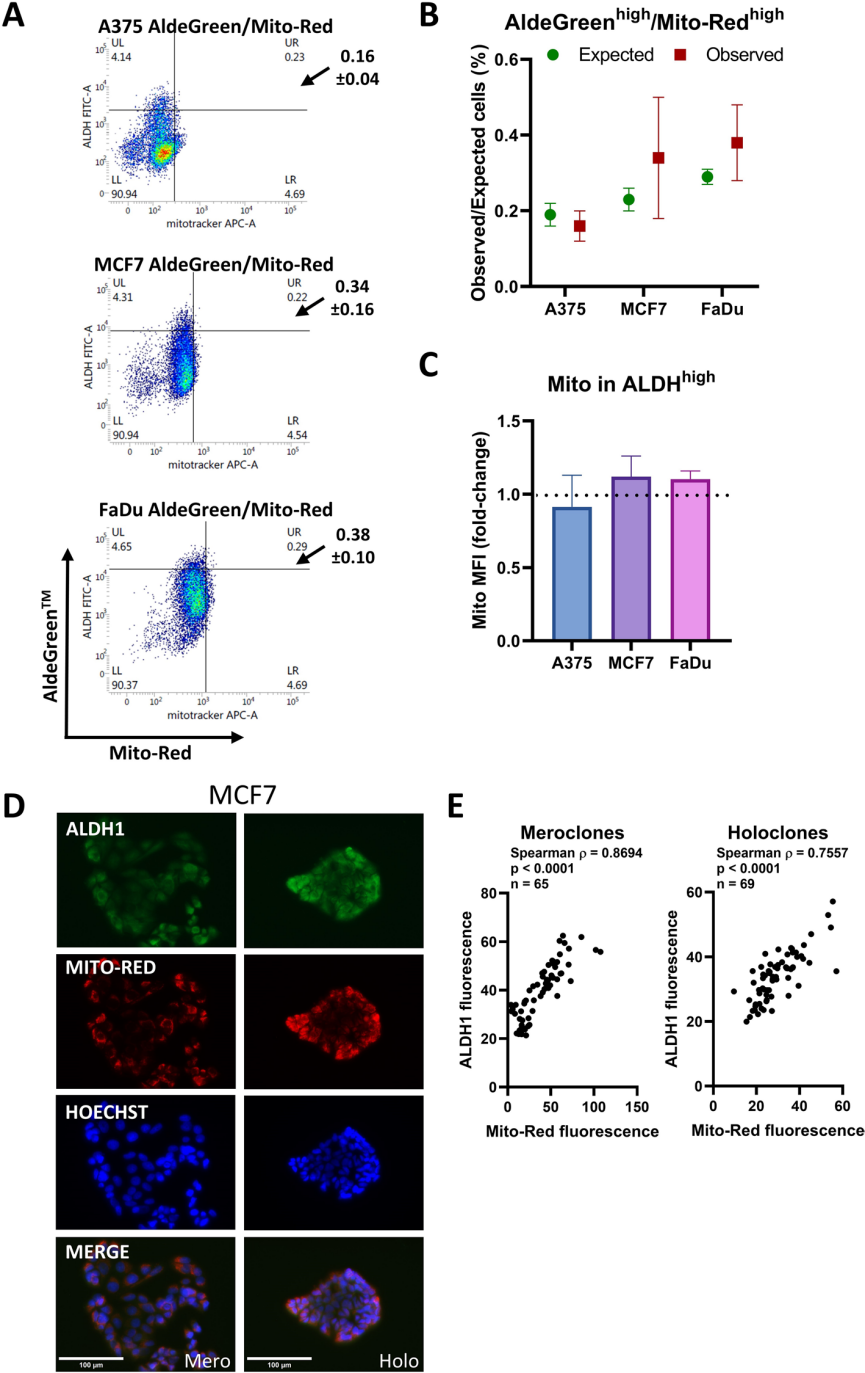
ALDH activity is not associated with mitochondrial membrane potential. **a** Flow cytometry detection of ALDH activity (AldeGreen) and mitochondrial membrane potential (Mito-Red) in A375, MCF7, and FaDu cells. The quadrants identify the top 5% of cells with high mitochondrial membrane potential and the highest 5% of AldeGreen. The values shown next to the graphs represent the mean ± SD from three independent experiments, whereas the numbers displayed in the quadrants correspond to the representative density plot shown in the figure. **b** Graphs show the expected percentage of cells in the region of interest if there is no relationship between the variables, compared with the observed percentage (mean ± SEM, *n* = 3 biological replicates). **c** The relative levels of Mito-Red (measured as MFI) in the top 5% of ALDH^+^ cells compared with the entire ALDH^+^ population across three cell lines. Values are normalized to the mean MFI of Mito-Red in the whole ALDH^+^ population for each respective cell line. The dashed horizontal line at 1.0 indicates no difference. **d** Representative images of MCF7 colonies stained with ALDH1 (green), Mito-Red (red), and Hoechst (blue). The scale bar represents 100 μm. Holo/mero/para clone next to the scale bar denotes the colony morphology type. **e** Fluorescence quantification of holoclones and meroclones using QuPath. Pairwise associations between markers were assessed using Spearman’s rank correlation. Correlation coefficients are reported in the graphs as Spearman’s *ρ* together with associated *p*-values. The number of analyzed cells is indicated in the graphs as *n*

To determine whether CSC (ALDH^high^) and non-CSC populations differ in higher or lower mitochondrial membrane potential, we compared mitochondrial membrane potential with ALDH activity (Fig. 4a; Supplementary Fig. S8). There were no significant associations of these two parameters in all three cancer cell lines using flow cytometry and gating of the AldeGreen^high^ population and Mito-Red^high^ cells (A375, *p* = 0.421; MCF7, *p* = 0.517; FaDu, *p* = 0.393) (Fig. 4b) or by comparing MFI values (Fig. 4c). Fluorescence staining of individual colonies (Fig. 4d; Supplementary Fig. S9A) showed correlations between mitochondrial membrane potential and ALDH1 in MCF7 cells (Spearman *ρ* = 0.8694, *p* < 0.0001 for meroclones and *ρ* = 0.7557, *p* < 0.0001 for holoclones) (Fig. 4e) and in FaDu cells (Spearman *ρ* = 0.6308, *p* < 0.0001 for meroclones and *ρ* = 0.4614, *p* < 0.0001 for holoclones) (Supplementary Fig. S9B). While flow cytometry analyses of subconfluent monolayer cultures did not reveal an association between AldeGreen^high^ and Mito-Red^high^ cells (Fig. 4a–c), imaging of clonally derived colonies did show a positive correlation (Fig. 4d, e). Notably, the different culture contexts and levels of cell–cell interactions between monolayers and colonies may account for this discrepancy.

To assess whether GLUT and mitochondrial membrane potential are affected by the cell cycle, we combined cell cycle profiling with Mito-Red and GLUT1. Specifically, we compared the distributions of cell cycle in the top 5% Mito-Red^high^ (Supplementary Fig. S10A) and GLUT^low^ populations (Supplementary Fig. S10B), showing that A375 and MCF7 cells with Mito-Red^high^ fluorescence were enriched in both G0/G1 and G2/M phase (upper left and upper right quadrant), whereas FaDu cells in G0/G1 displayed lower mitochondrial activity. GLUT1 staining revealed that GLUT1^low^ cells were more frequent in the G1 phase (lower left quadrant) compared with G2/M (lower right quadrant). These differences were not statistically significant. Other studies have shown shifts in mitochondrial membrane potential across cell cycle stages, such as increases from the early to late G1 phase [49] and transient depolarizations during mitosis [50]. Likewise, synchronized HeLa cells exhibit a metabolic shift: the G1 phase shows higher oxidative phosphorylation activity, while the S phase favors glycolysis [51]. However, CSCs are thought to show different cell cycle parameters than the tumor bulk, so it is not clear from these associations whether it is different cell cycle parameters that are responsible, or whether they are themselves caused by different CSC phenotypes.

In summary, these data show the associations between glucose uptake, mitochondrial activity assessed by mitochondrial membrane potential, and ALDH as a CSC marker. The data show that glucose uptake is not associated with reduced mitochondrial membrane potential, and that CSCs do not show reduced mitochondrial activity.

### Activity of LDH and SDH enzymes is variable regardless of clone morphology

**Fig. 5:**
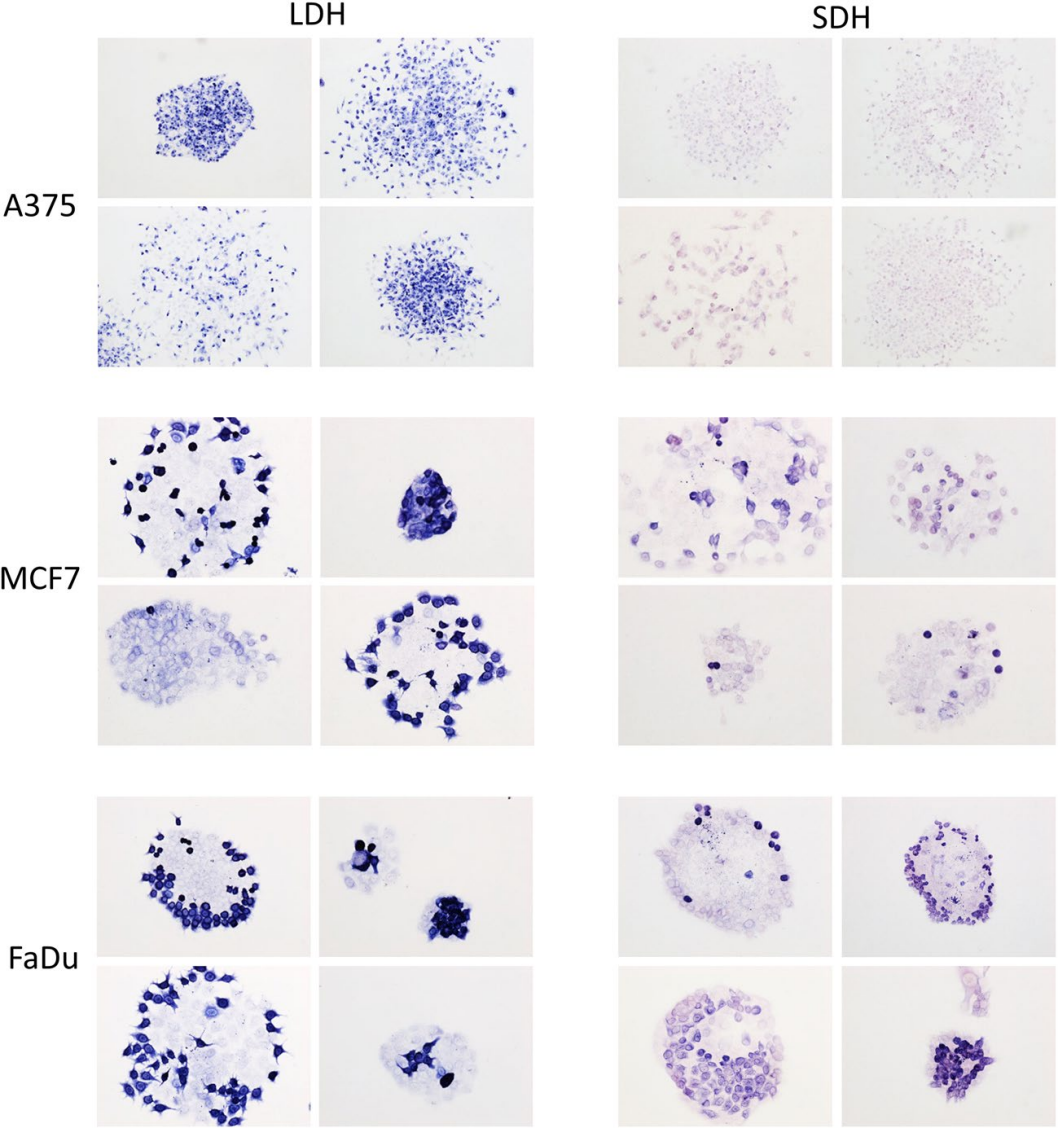
LDH and SDH enzyme activity is variable regardless of clone morphology. Representative images of LDH and SDH activity in A375, MCF7, and FaDu cells grown in low-density colonies (control: unsorted cells). Enzyme histochemistry was performed with NBT as a detection reagent (blue). Four separate colonies are shown for each enzyme in each cell line. Negative controls were prepared without substrate (data not shown)

Enzyme histochemistry was used to assess the relative levels of major enzymes involved in glycolysis (LDH) or the TCA cycle (SDH) in different colony types. LDH and SDH activity were variable in the three cell lines, with A375 showing very low SDH activity (Fig. 5). Both enzymes also showed substantial heterogeneity in individual cells, and showed strong staining of peripheral cells in some colonies, and of central cells in other colonies, with random distributions of staining of individual cells in other colonies (Fig. 5). These differences were not associated with colony morphology.

**Fig. 6:**
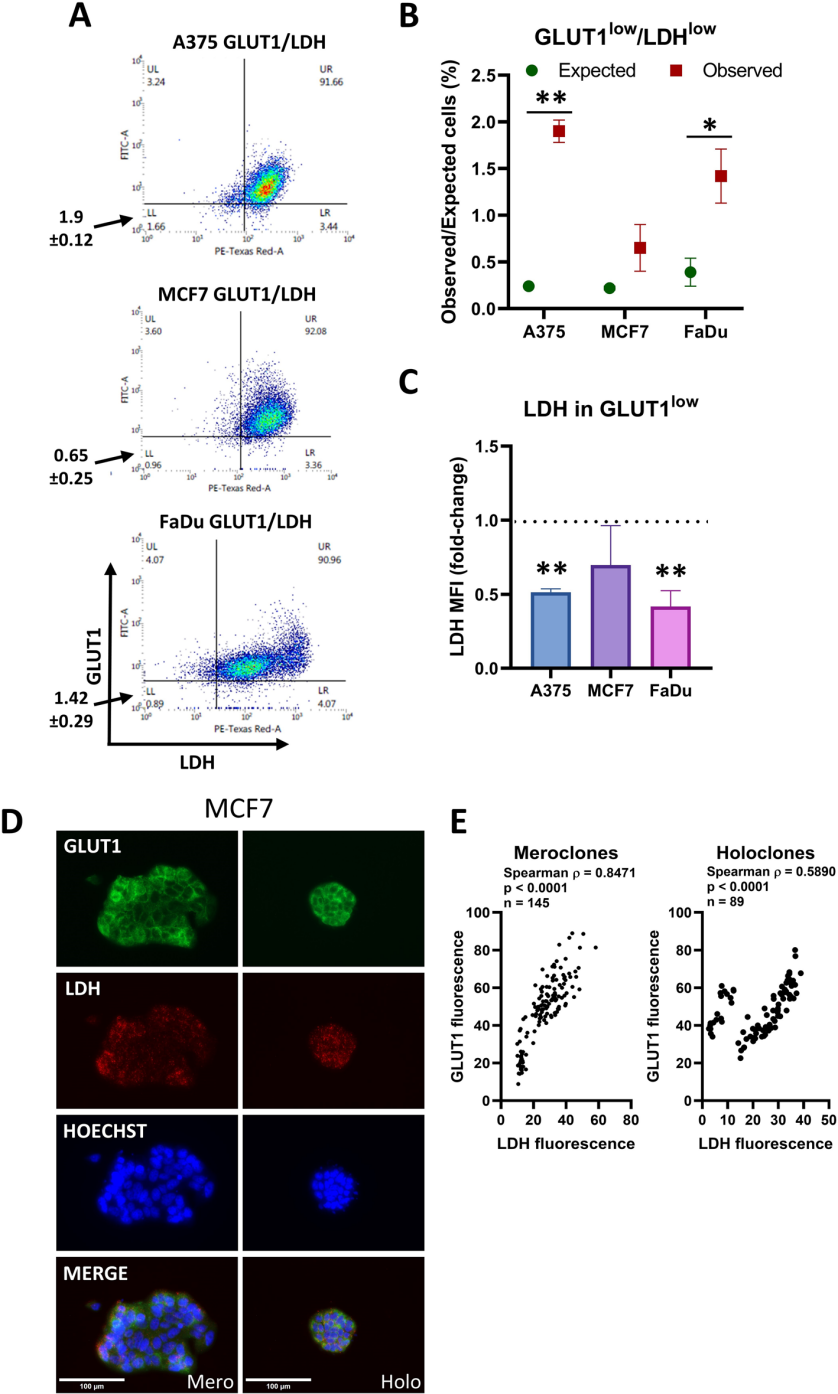
LDH activity correlates with GLUT1 levels. **a** Flow cytometry detection of LDH and glucose transporter in A375, MCF7, and FaDu cells. The quadrants identify cells in the lowest 5% of GLUT1 and the lowest 5% of LDH activity. The values shown next to the graphs represent the mean ± SD from three independent experiments, whereas the numbers displayed in the quadrants correspond to the representative density plot shown in the figure. **b** The expected percentage of cells in the region of interest if there is no relationship between the variables compared with the observed percentage (mean ± SEM, *n* = 3 biological replicates). *p < 0.05, **p < 0.01). **c** Relative LDH level (measured as MFI) in the lowest 5% of GLUT1^+^ cells compared with the entire GLUT1^+^ population across three cell lines. Values are normalized to the mean MFI of LDH in the whole GLUT1^+^ population for each respective cell line. The dashed horizontal line at 1.0 indicates no difference. Significant difference **p < 0.01. **d** Representative images of MCF7 colonies stained with GLUT1 (green), LDH (red), and Hoechst (blue). The scale bar represents 100 μm. Holo/mero/para clone next to the scale bar denotes the colony morphology type. **e** Fluorescence quantification of holoclones and meroclones using QuPath. Pairwise associations between markers were assessed using Spearman’s rank correlation. Correlation coefficients are reported as Spearman’s *ρ* together with associated *p*-values. The number of analyzed cells is indicated in as *n*

To assess the relationships of enzyme activities with other parameters, we employed the fluorescent substrate 5-cyano-2,3-di-(p-tolyl)tetrazolium chloride (CTC) in place of the colorimetric substrate NBT. Fluorescence microscopy of LDH activity revealed similar heterogeneity to the colorimetric assay (Supplementary Figs. 11–13). Quantification using flow cytometry showed that the populations of cells with low LDH activity were enriched for cells with low GLUT1 levels (A375, *p* = 0.006; MCF7, *p* = 0.224; FaDu, *p* = 0.033) (Fig. 6a, b; Supplementary Fig. S14). Analyzing the 5% of cells with the lowest GLUT1 levels showed that LDH was lower in this population in A375 (*p* < 0.01) and FaDu cells (*p* < 0.01), whereas MCF7 cells showed no significant change (Fig. 6c). Fluorescence staining in individual colonies (Fig. 6d; Supplementary Fig. S15A) showed a correlation between GLUT1 and LDH in MCF7 cells (Spearman *ρ* = 0.8471, *p* < 0.0001 for meroclones and *ρ* = 0.5890, *p* < 0.0001 for holoclones) (Fig. 6e) and in FaDu (Spearman *ρ* = 0.6280, *p* < 0.0001 for meroclones and *ρ* = 0.7499, *p* < 0.0001 for holoclones) (Supplementary Fig. 15b).

**Fig. 7:**
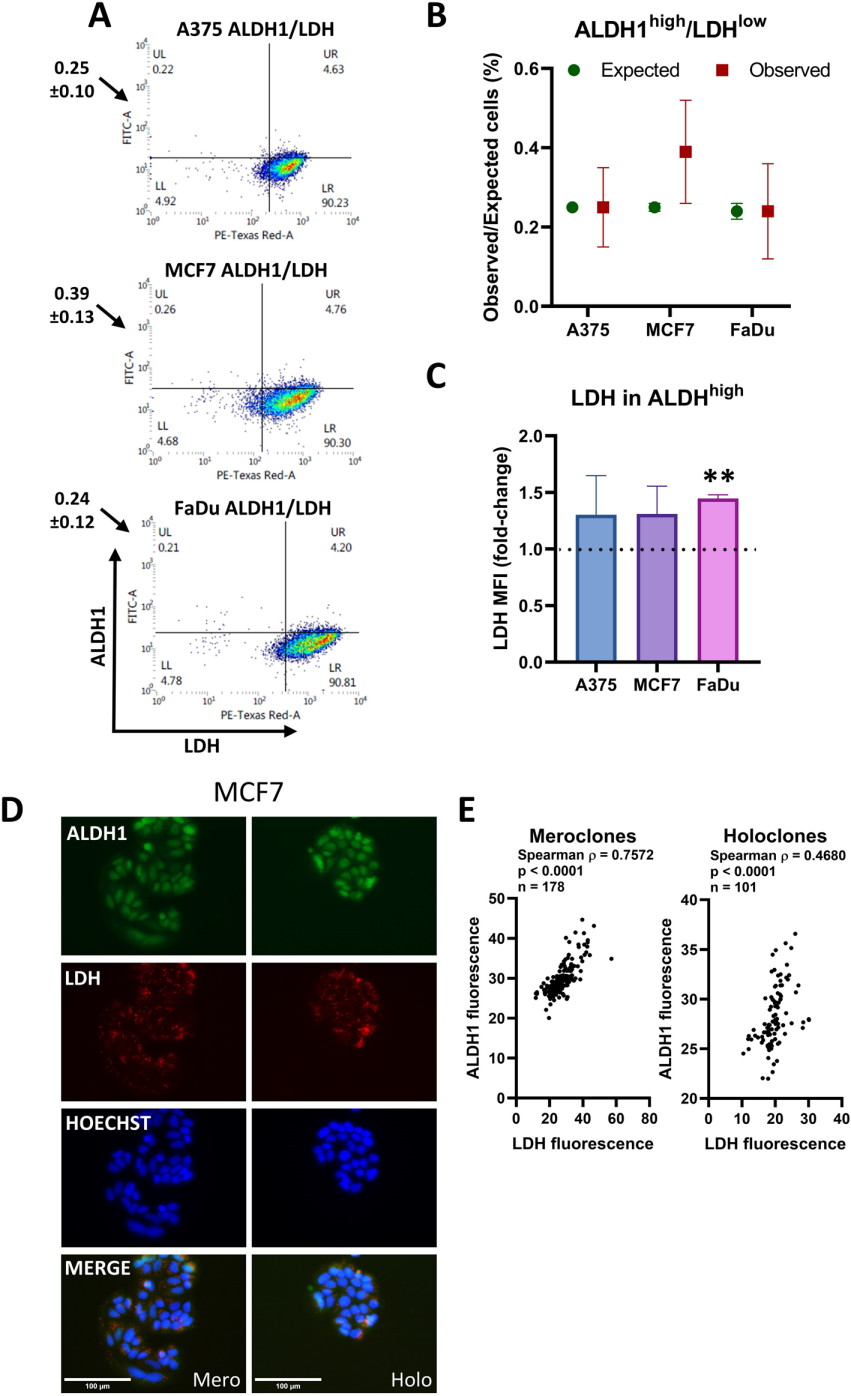
ALDH activity is not associated with LDH activity. **a** Flow cytometry detection of ALDH1 and LDH in A375, MCF7, and FaDu cells. The quadrants identify cells in the top 5% of ALDH activity and the lowest 5% of LDH activity. The values shown next to the graphs represent the mean ± SD from three independent experiments, whereas the numbers displayed in the quadrants correspond to the representative density plot shown in the figure. **b** The relative ratio of expected and observed cells in region of interest, if there is no relationship between the variables compared with the observed percentage (mean ± SEM, *n* = 3 biological replicates). **c** The relative level of LDH (measured as MFI) in the top 5% of ALDH^+^ cells compared with the entire ALDH^+^ population across three cell lines. Values are normalized to the mean MFI of LDH in the whole ALDH^+^ population for each respective cell line. The dashed horizontal line at 1.0 indicates no difference. **p < 0.01. **d** Representative images of MCF7 colonies stained with ALDH1 (green), LDH (red), and Hoechst (blue). The scale bar represents 100 μm. Holo/mero/para clone next to the scale bar denotes the colony morphology type. **e** Fluorescence quantification of holoclones and meroclones using QuPath. Pairwise associations between markers were assessed using Spearman’s rank correlation. Correlation coefficients are reported as Spearman’s *ρ* together with associated *p*-values. The number of analyzed cells is indicated as *n*

In the population of cells with low LDH there was no association with ALDH1 levels (A375, *p* = 0.984; MCF7, *p* = 0.362; FaDu, *p* = 0.956) using flow cytometry (Fig. 7a, b; Supplementary Fig. S16) or immunofluorescence (Fig. 7d; Supplementary Fig. S17). Fluorescence staining of colonies showed a correlation between ALDH and LDH in MCF7 cells (Spearman *ρ* = 0.7572, *p* < 0.0001 for meroclones and *ρ* = 0.4680, *p* < 0.0001 for holoclones) (Fig. 7e) and weaker correlations in FaDu cells (Spearman *ρ* = 0.2777, *p* < 0.05 for meroclones, and *ρ* = 0.3961, *p* < 0.0001 for holoclones) (Supplementary Fig. S17B).

Finally, we analyzed the numbers of strongly fluorescent cells in holoclones and meroclones, and assessed the mean ratio of cells with the top 5% fluorescence intensity for the indicated parameter in holoclones compared with meroclones in MCF7 and FaDu. The data show variability across different colonies without any statistically significant changes, except for LDH in FaDu cells, which showed a reduced number of LDH^high^ cells in meroclones (*p* < 0.01) (Supplementary Fig. S18).

### Metabolic phenotype exhibits plasticity

**Fig. 8:**
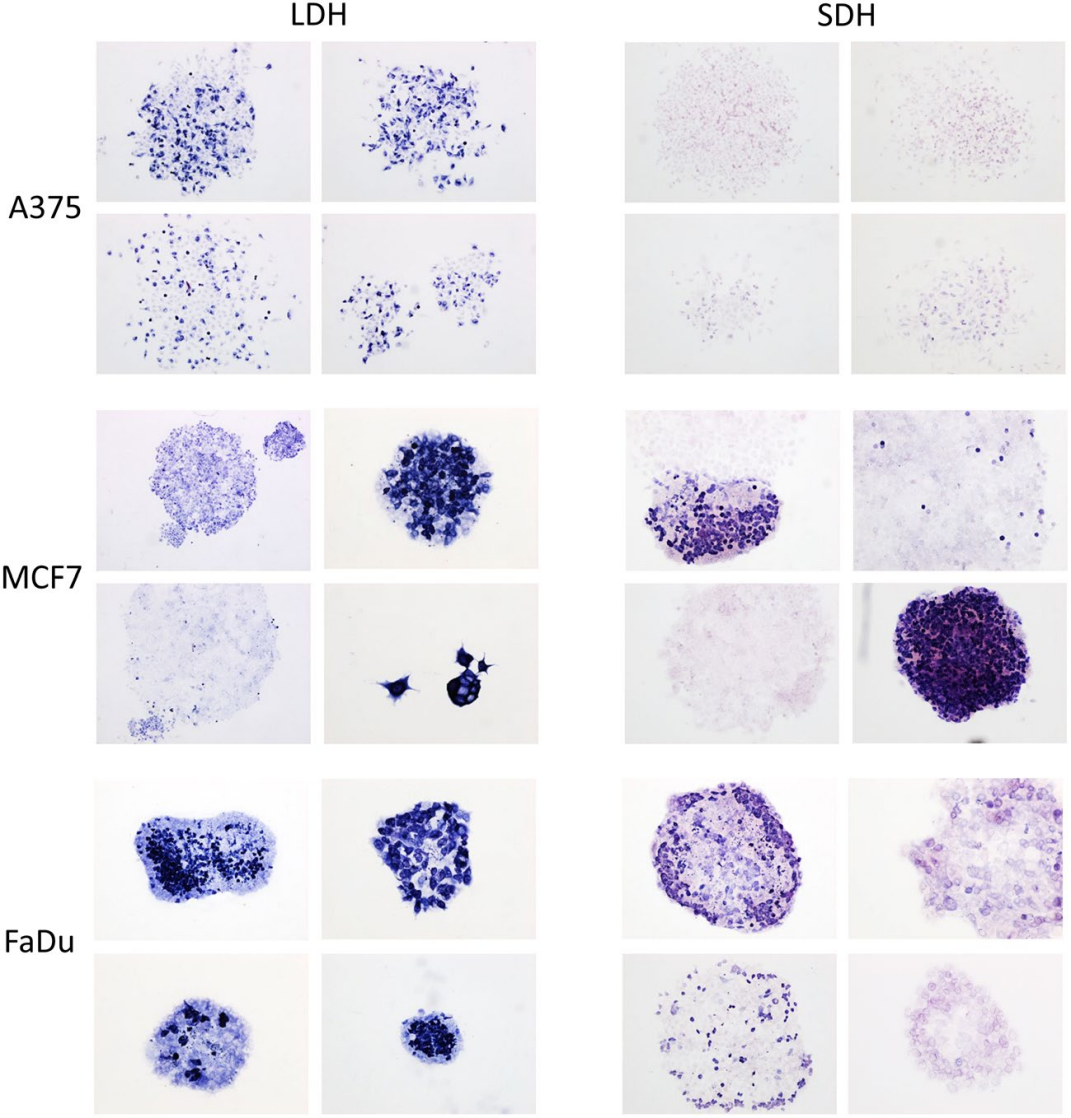
LDH and SDH activity is heterogeneous in ALDH^+^ sorted cells. Representative images of LDH and SDH activity in A375, MCF7, and FaDu cells grown in low density colonies after sorting the top 5% of ALDH^+^ cells. Enzyme histochemistry was performed with NBT as detection reagent (blue). Four separate colonies are shown for each enzyme in each cell line. Negative controls were prepared without substrate (data not shown)

To investigate whether metabolic phenotypes are hard-wired or plastic properties within cancer cell subpopulations, we used flow cytometry to isolate cells on the basis of their mitochondrial membrane potential or ALDH activity, and the sorted cells were plated at low density to form colonies for enzyme histochemistry. Colonies derived from Mito-Red^high^ cells showed similar distributions of LDH activity to unsorted cells, with heterogeneous levels in individual colonies and individual cells within a colony (Supplementary Fig. S19A, left panel). SDH activity also showed variation in individual colonies and in individual cells within colonies (Supplementary Fig. S19A, right panel). Similarly, colonies derived from ALDH^high^ cells showed both inter- and intra-heterogeneity of enzyme activities in colonies of all three cell lines (Fig. 8). Quantification and comparison of ALDH^high^ and Mito-Red^high^ sorted cells to unsorted cells showed no significant change (Supplementary Fig. S19B).

## Discussion

Cancer cells require increased bioenergetic, biosynthetic, and redox capacity to support their high rates of growth and proliferation. As part of their metabolic reprogramming, cancer cells show enhanced glycolysis even in the presence of sufficient oxygen, known as the Warburg effect. Although aerobic glycolysis is inefficient for energy production compared with mitochondrial OXPHOS, glycolytic intermediates serve to fulfill the increased demands for nucleic acid, lipid, and protein anabolism for biomass production in a growing tumor [52]. Thus, rapidly growing cancer cells require elevated uptake of available glucose, which is often associated with an increase in glucose transporters, particularly GLUT1 [19]. However, CSCs are relatively slow growing cells [1, 2], implying that they do not require such high rates of glucose uptake, glycolysis, and anabolic metabolism, but may rely more on OXPHOS for energy and metabolic homeostasis. Indeed, mitochondrial activities and OXPHOS are increased in CSC populations in a variety of cancer types, and inhibiting mitochondrial activity suppresses CSCs [53–56], although OXPHOS inhibition with increased fatty acid metabolism has also been associated with induction of the CSC phenotype in some situations [57]. Here, we set out to ask two principal questions: (1) Do cancer cells exhibit heterogeneity of glucose transport, glycolysis/OXPHOS, and mitochondrial membrane potential/mass? and (2) Do these parameters identify distinct cell populations that associate with CSC properties? We used cell lines for our studies, which have the advantage that all cells are exposed to an identical environment (availability of glucose, oxygen, and other nutrients), avoiding heterogeneity of metabolism in different areas of a tumor and thereby identifying intrinsic metabolic phenotypes. We also employed three cell lines representing three distinct common cancer types to investigate the commonality of metabolic phenotypes. A375, MCF-7, and FaDu are representative models of prevalent and widely studied human cancers—melanoma, luminal breast adenocarcinoma, and head-and-neck squamous cell carcinoma, respectively. Their popularity in research is not accidental: for instance, MCF-7 remains one of the most characterized and frequently used breast cancer lines owing to it representing the most common breast cancer subtype and its consistent tumorigenicity and hormone-responsiveness [58].

Previous studies have shown that somatic stem cells in many tissues have a distinct metabolic signature from progenitor cell populations that depends on limiting anabolism [14], and CSCs correspondingly show increased reliance on OXPHOS [55]. On the basis of this evidence that CSCs do not require higher than normal glucose uptake for anabolic pathways and show more mitochondrial OXPHOS than the tumor bulk, we assessed glucose transporter levels, glucose uptake, and mitochondrial mass and membrane potential in individual tumor cells. We also assessed glycolytic and TCA cycle enzyme activities at the single-cell level, and combined these assays to identify their relationships. In these analyses, we used a 5% cutoff to identify the subpopulation of cells with the trait that is purported to associate specifically with the CSC population of cancer cells. Cells outside that population were not further analyzed as they are classed as non-CSCs, which form the majority of the overall cancer. The 5% of cells enriched using one marker was then assessed to identify whether these cells are also enriched for the other marker. It is important to note that we did not employ broad correlations between markers across the entire cancer cell population in most analyses, we only correlated different characteristics within the putative CSC-enriched populations. It is also essential to point out that the 5% cutoff we used is imprecise, particularly for identifying different CSCs using different markers, and in different tumor cell lines. However, a cutoff approach is necessary for identifying CSCs and although variable, most tumors of different origins are estimated to contain between 2% and 10% CSCs [4, 38, 39, 59], making 5% a rational and practical value to allow significant enrichment of the CSC population and remove all or the majority of non-CSCs prior to statistical analysis [6].

The first major finding of our work is that all of these parameters show marked heterogeneity, with 100-fold or more variance in individual cells in each cell line. The second important finding is that glucose uptake and GLUT1 levels are positively correlated with mitochondrial membrane potential in individual tumor cells. Thirdly, we found that holoclones, representing a stem cell population, have higher levels of GLUT1 than meroclones or paraclones. Holoclones of FaDu cells, representing a stem cell population, showed a 1.75-fold higher percentage of GLUT1^high^ cells than meroclones (Supplementary Fig. S18), although this did not reach statistical significance (*p* = 0.09) owing to the relatively small number of cells analyzed compared with flow cytometry. Squamous cancer CSCs showing higher GLUT1 is consistent with gene expression profiling of holoclones derived from normal human skin keratinocytes [60], and indicates that high GLUT1 levels correspond with a CSC phenotype. Notably, this increased glucose transport correlates with high ALDH, a commonly used CSC marker that identifies a specific subtype of “epithelial-like” CSCs rather than identifying all CSCs in a tumor [8, 61]. Previous studies showed that ALDH^+^ CSCs are less glycolytic compared with the majority of cells in the tumor cell bulk, and compared with other CSC populations in the same tumors [62]. However, different epithelial-like or mesenchymal-like CSC subtypes of the same tumor cell line show different glucose uptake and mitochondrial activity [63]. Recently, we have shown that CSCs identified by low proteasome activity are associated with the population identified by high ALDH activity, and that these CSCs have a low level of glucose transporters [64].

We also found that high glucose transport associates with high mitochondrial membrane potential as a marker of OXPHOS activity, and with a higher level of LDH activity as a marker of glycolysis. Therefore, tumor cells with high glucose uptake required for Warburg metabolism show increased, rather than decreased, mitochondrial activity, indicating that these alternative metabolic pathways are not necessarily inversely associated in cancer, and tumor cells may show both increased glycolysis and increased mitochondrial activity. Again, although many studies have concentrated on either aerobic glycolysis or mitochondrial OXPHOS as being associated with CSCs, our study indicates an increase of both and suggest a mixed phenotype seen previously [65]. Thus, the hypothesis that cells balance their carbon metabolism through glycolysis or OXPHOS, with one predominating at the expense of the other, is untrue in all cancer cells. As such, these cells are expected to show sensitivity to both glycolytic and mitochondrial inhibition.

## Conclusions

Metabolic heterogeneity and plasticity in cancer cells are well established in literature [21, 66–71], allowing us to investigate their associations with CSC properties. On the basis that commonly used CSC markers are not universal, and previous observations that normal tissue stem cells and CSCs may show unique metabolic profiles, we set out to examine the heterogeneity of tumor cell metabolism and to cross-correlate these with each other and with CSC markers. We uncovered a high degree of glucose metabolism variability in individual cancer cells, with a correlation identified between high mitochondrial membrane potential and high levels of glucose transport. These features correlate with stem cells assessed by holoclone formation but are not consistently observed in the ALDH^+^ CSC population. Overall, the data add to the growing realization of tumor cell metabolic heterogeneity and plasticity, and do not support the hypothesis that CSCs universally exhibit a specific metabolic phenotype. These findings are important not only for a fuller understanding of cellular metabolism and heterogeneity in cancer but also for an improved understanding of the potential therapeutic effects of glucose metabolism inhibitors on CSC and tumor bulk cell populations.

## Supplementary Information

Additional file 1.

## Data Availability

The data produced in this work are contained with the manuscript and supplementary information.

## Abbreviations

ALDH: Aldehyde dehydrogenase
CSC: Cancer stem-like cell
GLUT: Glucose transporter
IDH: Isocitrate dehydrogenase
LDH: Lactate dehydrogenase
MFI: Mean fluorescence intensity
OXPHOS: Oxidative phosphorylation
SDH: Succinate dehydrogenase
TCA: Tricarboxylic acid cycle

## References

[1] Clarke MF. Clinical and therapeutic implications of cancer stem cells. N Engl J Med. 2019;380(23):2237–45. 31167052 10.1056/NEJMra1804280

[2] Batlle E, Clevers H. Cancer stem cells revisited. Nat Med. 2017;23(10):1124–34. 28985214 10.1038/nm.4409

[3] Maitland NJ, Collins AT. Prostate cancer stem cells: a new target for therapy. J Clin Oncol. 2008;26(17):2862–70. 18539965 10.1200/JCO.2007.15.1472

[4] Ishizawa K, Rasheed ZA, Karisch R, Wang Q, Kowalski J, Susky E, et al. Tumor-initiating cells are rare in many human tumors. Cell Stem Cell. 2010;7(3):279–82. 20804964 10.1016/j.stem.2010.08.009 PMC2945729

[5] Klonisch T, Wiechec E, Hombach-Klonisch S, Ande SR, Wesselborg S, Schulze-Osthoff K, et al. Cancer stem cell markers in common cancers-therapeutic implications. Trends Mol Med. 2008;14(10):450–60. 18775674 10.1016/j.molmed.2008.08.003

[6] Liu Y, Nenutil R, Appleyard MV, Murray K, Boylan M, Thompson AM, et al. Lack of correlation of stem cell markers in breast cancer stem cells. Br J Cancer. 2014;110(8):2063–71. 24577057 10.1038/bjc.2014.105 PMC3992489

[7] Kim J, Villadsen R, Sørlie T, Fogh L, Grønlund SZ, Fridriksdottir AJ, et al. Tumor initiating but differentiated luminal-like breast cancer cells are highly invasive in the absence of basal-like activity. Proc Natl Acad Sci USA. 2012;109(16):6124–9. 22454501 10.1073/pnas.1203203109 PMC3341000

[8] Liu S, Cong Y, Wang D, Sun Y, Deng L, Liu Y, et al. Breast cancer stem cells transition between epithelial and mesenchymal states reflective of their normal counterparts. Stem Cell Rep. 2014;2(1):78–91. 10.1016/j.stemcr.2013.11.009 PMC3916760 24511467

[9] Prasetyanti PR, Medema JP. Intra-tumor heterogeneity from a cancer stem cell perspective. Mol Cancer. 2017;16(1):41. 28209166 10.1186/s12943-017-0600-4 PMC5314464

[10] Lenos KJ, Miedema DM, Lodestijn SC, Nijman LE, van den Bosch T, Romero Ros X, et al. Stem cell functionality is microenvironmentally defined during tumour expansion and therapy response in colon cancer. Nat Cell Biol. 2018;20(10):1193–202. 30177776 10.1038/s41556-018-0179-z PMC6163039

[11] Gola A, Fuchs E. Environmental control of lineage plasticity and stem cell memory. Curr Opin Cell Biol. 2021;69:88–95. 33535130 10.1016/j.ceb.2020.12.015 PMC8058249

[12] Beaver CM, Ahmed A, Masters JR. Clonogenicity: holoclones and meroclones contain stem cells. PLoS ONE. 2014;9(2):e89834. 24587067 10.1371/journal.pone.0089834 PMC3935944

[13] Barrandon Y, Green H. Three clonal types of keratinocyte with different capacities for multiplication. Proc Natl Acad Sci USA. 1987;84(8):2302–6. 2436229 10.1073/pnas.84.8.2302 PMC304638

[14] Meacham CE, DeVilbiss AW, Morrison SJ. Metabolic regulation of somatic stem cells in vivo. Nat Rev Mol Cell Biol. 2022;23(6):428–43. 35228719 10.1038/s41580-022-00462-1

[15] Hay N. Reprogramming glucose metabolism in cancer: can it be exploited for cancer therapy? Nat Rev Cancer. 2016;16(10):635–49. 27634447 10.1038/nrc.2016.77 PMC5516800

[16] Pavlova NN, Zhu J, Thompson CB. The hallmarks of cancer metabolism: still emerging. Cell Metab. 2022;34(3):355–77. 35123658 10.1016/j.cmet.2022.01.007 PMC8891094

[17] Liberti MV, Locasale JW. The warburg effect: how does it benefit cancer cells? Trends Biochem Sci. 2016;41(3):211–8. 26778478 10.1016/j.tibs.2015.12.001 PMC4783224

[18] Adekola K, Rosen ST, Shanmugam M. Glucose transporters in cancer metabolism. Curr Opin Oncol. 2012;24(6):650–4. 22913968 10.1097/CCO.0b013e328356da72 PMC6392426

[19] Szablewski L. Expression of glucose transporters in cancers. Biochimica et Biophysica Acta (BBA). 2013;1835(2):164–9. 23266512 10.1016/j.bbcan.2012.12.004

[20] Pavlova NN, Thompson CB. The emerging hallmarks of cancer metabolism. Cell Metab. 2016;23(1):27–47. 26771115 10.1016/j.cmet.2015.12.006 PMC4715268

[21] Dupuy F, Tabariès S, Andrzejewski S, Dong Z, Blagih J, Annis MG, et al. PDK1-dependent metabolic reprogramming dictates metastatic potential in breast cancer. Cell Metab. 2015;22(4):577–89. 26365179 10.1016/j.cmet.2015.08.007

[22] Thoudam T, Chanda D, Sinam IS, Kim BG, Kim MJ, Oh CJ, et al. Noncanonical PDK4 action alters mitochondrial dynamics to affect the cellular respiratory status. Proc Natl Acad Sci USA. 2022;119(34):e2120157119. 35969774 10.1073/pnas.2120157119 PMC9407676

[23] Nguyen NTB, Gevers S, Kok RNU, Burgering LM, Neikes H, Akkerman N, et al. Lactate controls cancer stemness and plasticity through epigenetic regulation. Cell Metab. 2025;37(4):903-919.e10. 39933514 10.1016/j.cmet.2025.01.002

[24] Banerjee A, Arvinrad P, Darley M, Laversin SA, Parker R, Rose-Zerilli MJJ, et al. The effects of restricted glycolysis on stem-cell like characteristics of breast cancer cells. Oncotarget. 2018;9(33):23274–88. 29796188 10.18632/oncotarget.25299 PMC5955399

[25] Farnie G, Sotgia F, Lisanti MP. High mitochondrial mass identifies a sub-population of stem-like cancer cells that are chemo-resistant. Oncotarget. 2015;6(31):30472–86. 26421710 10.18632/oncotarget.5401 PMC4741545

[26] Peiris-Pagès M, Martinez-Outschoorn UE, Pestell RG, Sotgia F, Lisanti MP. Cancer stem cell metabolism. Breast Cancer Res. 2016;18(1):55. 27220421 10.1186/s13058-016-0712-6 PMC4879746

[27] Vlashi E, Lagadec C, Vergnes L, Reue K, Frohnen P, Chan M, et al. Metabolic differences in breast cancer stem cells and differentiated progeny. Breast Cancer Res Treat. 2014;146(3):525–34. 25007966 10.1007/s10549-014-3051-2 PMC4131557

[28] Ye XQ, Li Q, Wang GH, Sun FF, Huang GJ, Bian XW, et al. Mitochondrial and energy metabolism-related properties as novel indicators of lung cancer stem cells. Int J Cancer. 2011;129(4):820–31. 21520032 10.1002/ijc.25944

[29] Sukumar M, Liu J, Mehta GU, Patel SJ, Roychoudhuri R, Crompton JG, et al. Mitochondrial membrane potential identifies cells with enhanced stemness for cellular therapy. Cell Metab. 2016;23(1):63–76. 26674251 10.1016/j.cmet.2015.11.002 PMC4747432

[30] Courtois S, Durán RV, Giraud J, Sifré E, Izotte J, Mégraud F, et al. Metformin targets gastric cancer stem cells. Eur J Cancer. 2017;84:193–201. 28822889 10.1016/j.ejca.2017.07.020

[31] Hirsch HA, Iliopoulos D, Tsichlis PN, Struhl K. Metformin selectively targets cancer stem cells, and acts together with chemotherapy to block tumor growth and prolong remission. Cancer Res. 2009;69(19):7507–11. 19752085 10.1158/0008-5472.CAN-09-2994 PMC2756324

[32] Janzer A, German NJ, Gonzalez-Herrera KN, Asara JM, Haigis MC, Struhl K. Metformin and phenformin deplete tricarboxylic acid cycle and glycolytic intermediates during cell transformation and NTPs in cancer stem cells. Proc Natl Acad Sci USA. 2014;111(29):10574–9. 25002509 10.1073/pnas.1409844111 PMC4115496

[33] Lonardo E, Cioffi M, Sancho P, Sanchez-Ripoll Y, Trabulo SM, Dorado J, et al. Metformin targets the metabolic Achille’s heel of human pancreatic cancer stem cells. PLoS ONE. 2013;8(10):e76518. 24204632 10.1371/journal.pone.0076518 PMC3799760

[34] Brown JR, Chan DK, Shank JJ, Griffith KA, Fan H, Szulawski R, et al. Phase II clinical trial of metformin as a cancer stem cell-targeting agent in ovarian cancer. JCI Insight. 2020;5(11):e133247. 32369446 10.1172/jci.insight.133247 PMC7308054

[35] Pajak B, Siwiak E, Sołtyka M, Priebe A, Zieliński R, Fokt I, et al. 2-deoxy-D-glucose and its analogs: from diagnostic to therapeutic agents. Int J Mol Sci. 2019;21(1):234. 31905745 10.3390/ijms21010234 PMC6982256

[36] Schmidt MC, O’Donnell AF. ‘Sugarcoating’ 2-deoxyglucose: mechanisms that suppress its toxic effects. Curr Genet. 2021;67(1):107–14. 33136227 10.1007/s00294-020-01122-7 PMC7886833

[37] Zhang D, Li J, Wang F, Hu J, Wang S, Sun Y. 2-deoxy-D-glucose targeting of glucose metabolism in cancer cells as a potential therapy. Cancer Lett. 2014;355(2):176–83. 25218591 10.1016/j.canlet.2014.09.003

[38] Harper LJ, Piper K, Common J, Fortune F, Mackenzie IC. Stem cell patterns in cell lines derived from head and neck squamous cell carcinoma. J Oral Pathol Med. 2007;36(10):594–603. 17944752 10.1111/j.1600-0714.2007.00617.x

[39] Ginestier C, Hur MH, Charafe-Jauffret E, Monville F, Dutcher J, Brown M, et al. ALDH1 is a marker of normal and malignant human mammary stem cells and a predictor of poor clinical outcome. Cell Stem Cell. 2007;1(5):555–67. 18371393 10.1016/j.stem.2007.08.014 PMC2423808

[40] Visvader JE, Lindeman GJ. Cancer stem cells in solid tumours: accumulating evidence and unresolved questions. Nat Rev Cancer. 2008;8(10):755–68. 18784658 10.1038/nrc2499

[41] Van Noorden CJF. Imaging enzymes at work: metabolic mapping by enzyme histochemistry. J Histochem Cytochem. 2010;58(6):481–97. 20124092 10.1369/jhc.2010.955518 PMC2874181

[42] Lee KS, Su X, Huan T. Metabolites are not genes—avoiding the misuse of pathway analysis in metabolomics. Nat Metab. 2025;7(5):858–61. 40211046 10.1038/s42255-025-01283-0

[43] Schatton D, Rugarli EI. A concert of RNA-binding proteins coordinates mitochondrial function. Crit Rev Biochem Mol Biol. 2018;53(6):652–66. 30741581 10.1080/10409238.2018.1553927

[44] Ni X, Lu CP, Xu GQ, Ma JJ. Transcriptional regulation and post-translational modifications in the glycolytic pathway for targeted cancer therapy. Acta Pharmacol Sin. 2024;45(8):1533–55. 38622288 10.1038/s41401-024-01264-1 PMC11272797

[45] Xie AX, Tansey W, Reznik E. UnitedMet harnesses RNA-metabolite covariation to impute metabolite levels in clinical samples. Nat Cancer. 2025;6(5):892–906. 40251399 10.1038/s43018-025-00943-0 PMC12122372

[46] Yamada K, Saito M, Matsuoka H, Inagaki N. A real-time method of imaging glucose uptake in single, living mammalian cells. Nat Protoc. 2007;2(3):753–62. 17406637 10.1038/nprot.2007.76

[47] Locke M, Heywood M, Fawell S, Mackenzie IC. Retention of intrinsic stem cell hierarchies in carcinoma-derived cell lines. Cancer Res. 2005;65(19):8944–50. 16204067 10.1158/0008-5472.CAN-05-0931

[48] Poot M, Zhang YZ, Krämer JA, Wells KS, Jones LJ, Hanzel DK, et al. Analysis of mitochondrial morphology and function with novel fixable fluorescent stains. J Histochem Cytochem. 1996;44(12):1363–72. 8985128 10.1177/44.12.8985128

[49] Schieke SM, McCoy JP, Finkel T. Coordination of mitochondrial bioenergetics with G1 phase cell cycle progression. Cell Cycle. 2008;7(12):1782–7. 18583942 10.4161/cc.7.12.6067 PMC3399174

[50] Hirusaki K, Yokoyama K, Cho K, Ohta Y. Temporal depolarization of mitochondria during M phase. Sci Rep. 2017;7(1):16044. 29167496 10.1038/s41598-017-15907-3 PMC5700041

[51] Liu J, Peng Y, Shi L, Wan L, Inuzuka H, Long J, et al. Skp2 dictates cell cycle-dependent metabolic oscillation between glycolysis and TCA cycle. Cell Res. 2021;31(1):80–93. 32669607 10.1038/s41422-020-0372-z PMC7852548

[52] DeBerardinis RJ, Chandel NS. Fundamentals of cancer metabolism. Sci Adv. 2016;2(5):e1600200. 27386546 10.1126/sciadv.1600200 PMC4928883

[53] Sighel D, Notarangelo M, Aibara S, Re A, Ricci G, Guida M, et al. Inhibition of mitochondrial translation suppresses glioblastoma stem cell growth. Cell Rep. 2021;35(4):109024. 33910005 10.1016/j.celrep.2021.109024 PMC8097689

[54] Nimmakayala RK, Rauth S, Chirravuri Venkata R, Marimuthu S, Nallasamy P, Vengoji R, et al. PGC1α-mediated metabolic reprogramming drives the stemness of pancreatic precursor lesions. Clin Cancer Res. 2021;27(19):5415–29. 34172498 10.1158/1078-0432.CCR-20-5020 PMC8709878

[55] Berlin C, Mauerer B, Cauchy P, Luenstedt J, Sankowski R, Marx L, et al. Single-cell deconvolution reveals high lineage- and location-dependent heterogeneity in mesenchymal multivisceral stage 4 colorectal cancer. J Clin Invest. 2024;134(5):e169576. 10.1172/JCI169576 PMC10904044 38153787

[56] Noh JK, Woo SR, Kong M, Lee MK, Lee JW, Lee YC, et al. Gene signature predicting recurrence in oral squamous cell carcinoma is characterized by increased oxidative phosphorylation. Mol Oncol. 2023;17(1):134–49. 36271693 10.1002/1878-0261.13328 PMC9812830

[57] Chen CL, Uthaya Kumar DB, Punj V, Xu J, Sher L, Tahara SM, et al. NANOG metabolically reprograms tumor-initiating stem-like cells through tumorigenic changes in oxidative phosphorylation and fatty acid metabolism. Cell Metab. 2016;23(1):206–19. 26724859 10.1016/j.cmet.2015.12.004 PMC4715587

[58] Holliday DL, Speirs V. Choosing the right cell line for breast cancer research. Breast Cancer Res. 2011;13(4):215. 21884641 10.1186/bcr2889 PMC3236329

[59] dos Santos RV, da Silva LM. A possible explanation for the variable frequencies of cancer stem cells in tumors. PLoS ONE. 2013;8(8):e69131. 23950884 10.1371/journal.pone.0069131 PMC3737222

[60] Ali D, Alhattab D, Jafar H, Alzubide M, Sharar N, Bdour S, et al. Differential marker expression between keratinocyte stem cells and their progeny generated from a single colony. Int J Mol Sci. 2021;22(19):10810. 34639148 10.3390/ijms221910810 PMC8509450

[61] Ricardo S, Vieira AF, Gerhard R, Leitao D, Pinto R, Cameselle-Teijeiro JF, et al. Breast cancer stem cell markers CD44, CD24 and ALDH1: expression distribution within intrinsic molecular subtype. J Clin Pathol. 2011;64(11):937–46. 21680574 10.1136/jcp.2011.090456

[62] Nimmakayala RK, Leon F, Rachagani S, Rauth S, Nallasamy P, Marimuthu S, et al. Metabolic programming of distinct cancer stem cells promotes metastasis of pancreatic ductal adenocarcinoma. Oncogene. 2021;40(1):215–31. 33110235 10.1038/s41388-020-01518-2 PMC10041665

[63] Gammon L, Biddle A, Heywood HK, Johannessen AC, Mackenzie IC. Sub-sets of cancer stem cells differ intrinsically in their patterns of oxygen metabolism. PLoS ONE. 2013;8(4):e62493. 23638097 10.1371/journal.pone.0062493 PMC3640080

[64] Krkoška M, Tylichová Z, Zatloukalová P, Müller P, Vojtěšek B, Coates PJ. Heterogeneous protein dynamics links to mitochondrial activity, glucose transporter, and ALDH cancer stem cell properties. BMC Cancer. 2025;25(1):1085. 40597978 10.1186/s12885-025-14460-x PMC12210997

[65] Liu CC, Chou KT, Hsu JW, Lin JH, Hsu TW, Yen DHT, et al. High metabolic rate and stem cell characteristics of esophageal cancer stem-like cells depend on the Hsp27-AKT-HK2 pathway. Int J Cancer. 2019;145(8):2144–56. 30920655 10.1002/ijc.32301

[66] Kondo H, Ratcliffe CDH, Hooper S, Ellis J, MacRae JI, Hennequart M, et al. Single-cell resolved imaging reveals intra-tumor heterogeneity in glycolysis, transitions between metabolic states, and their regulatory mechanisms. Cell Rep. 2021;34(7):108750. 33596424 10.1016/j.celrep.2021.108750 PMC7900713

[67] Reinfeld BI, Madden MZ, Wolf MM, Chytil A, Bader JE, Patterson AR, et al. Cell-programmed nutrient partitioning in the tumour microenvironment. Nature. 2021;593(7858):282–8. 33828302 10.1038/s41586-021-03442-1 PMC8122068

[68] Hensley CT, Faubert B, Yuan Q, Lev-Cohain N, Jin E, Kim J, et al. Metabolic heterogeneity in human lung tumors. Cell. 2016;164(4):681–94. 26853473 10.1016/j.cell.2015.12.034 PMC4752889

[69] Fendt SM, Frezza C, Erez A. Targeting metabolic plasticity and flexibility dynamics for cancer therapy. Cancer Discov. 2020;10(12):1797–807. 33139243 10.1158/2159-8290.CD-20-0844 PMC7710573

[70] McGuirk S, Audet-Delage Y, St-Pierre J. Metabolic fitness and plasticity in cancer progression. Trends Cancer. 2020;6(1):49–61. 31952781 10.1016/j.trecan.2019.11.009

[71] DeBerardinis RJ, Chandel NS. We need to talk about the Warburg effect. Nat Metab. 2020;2(2):127–9. 32694689 10.1038/s42255-020-0172-2

