# Supplementary material for: Heterogeneity of glucose metabolism and uptake identifies distinct cancer cell and cancer stem cell phenotypes
Source: Cell Mol Biol Lett. 2026 Jan 17;31:8. doi: 10.1186/s11658-025-00837-0 (PMC12829025; doi:10.1186/s11658-025-00837-0)
Supplement: Supplementary file 1 — Additional file 1. [file 11658_2025_837_MOESM1_ESM.pdf]

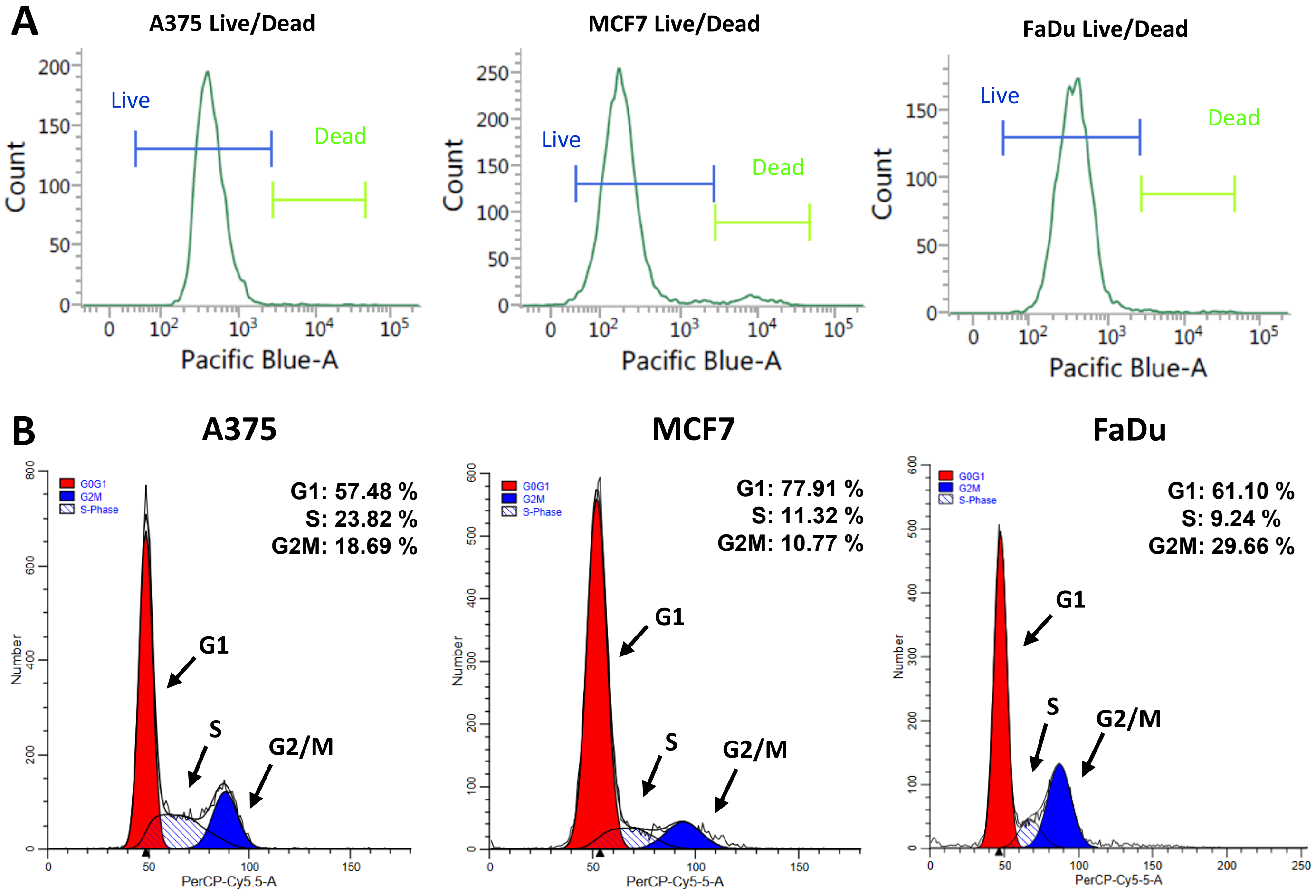

Suppl. Figure 1: Viability and cell cycle in A375, MCF7 and FaDu cells
(A) Flow cytometry detection of viability (Live/Dead staining) in control untreated A375, MCF7 and FaDu cells. **(B)** Flow cytometry detection of cell cycle in control untreated A375, MCF7 and FaDu cells. Cell cycle distribution was analyzed using ModFit software. Histograms are representatives from three independent experiments.

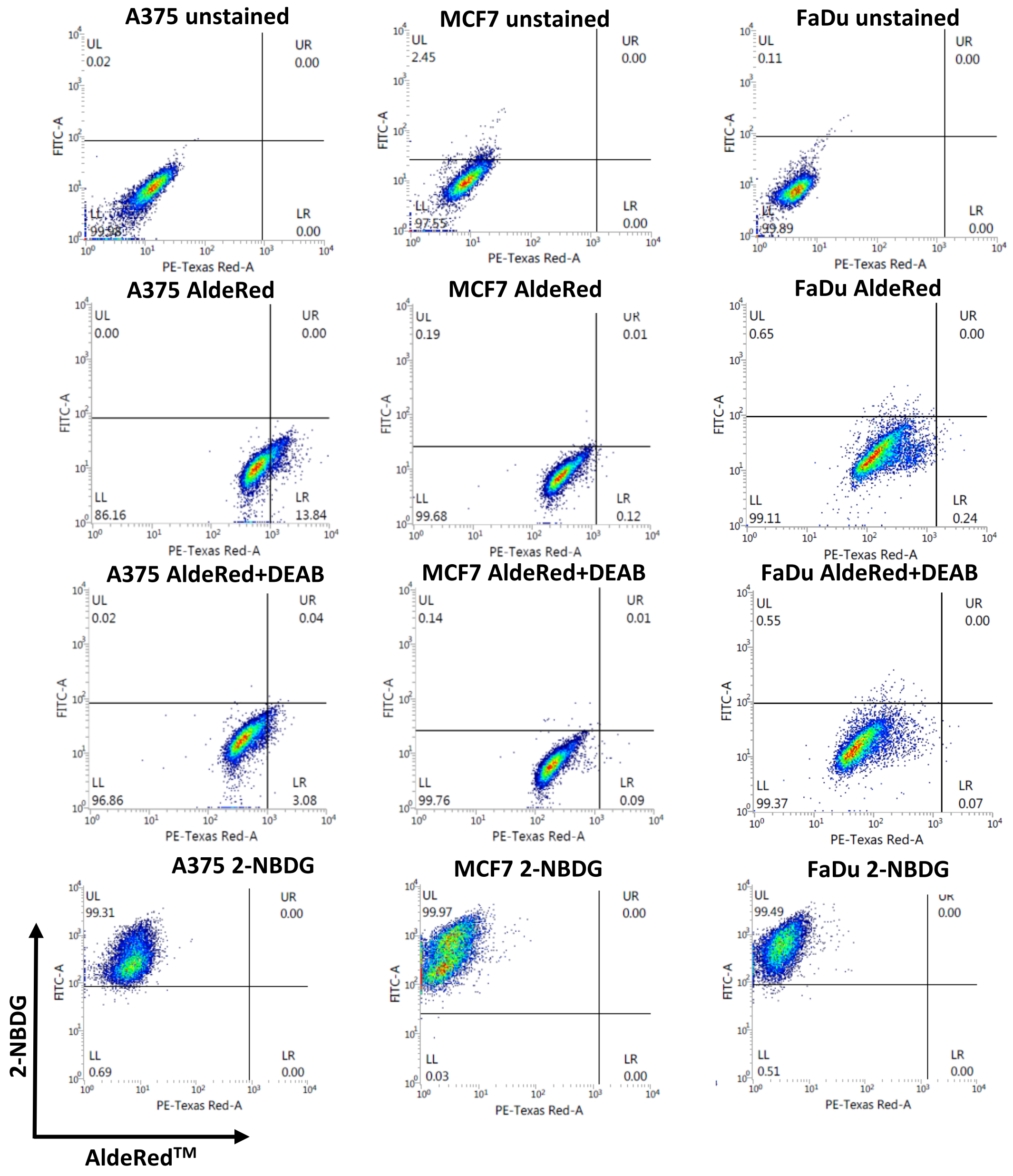

Suppl. Figure 2: ALDH activity vs glucose transport in A375, MCF7 and FaDu cells

Glucose uptake (2-NBDG) and ALDH1 activity (AldeRed) in A375, MCF7 and FaDu cells. Density plots are representatives from three independent experiments.

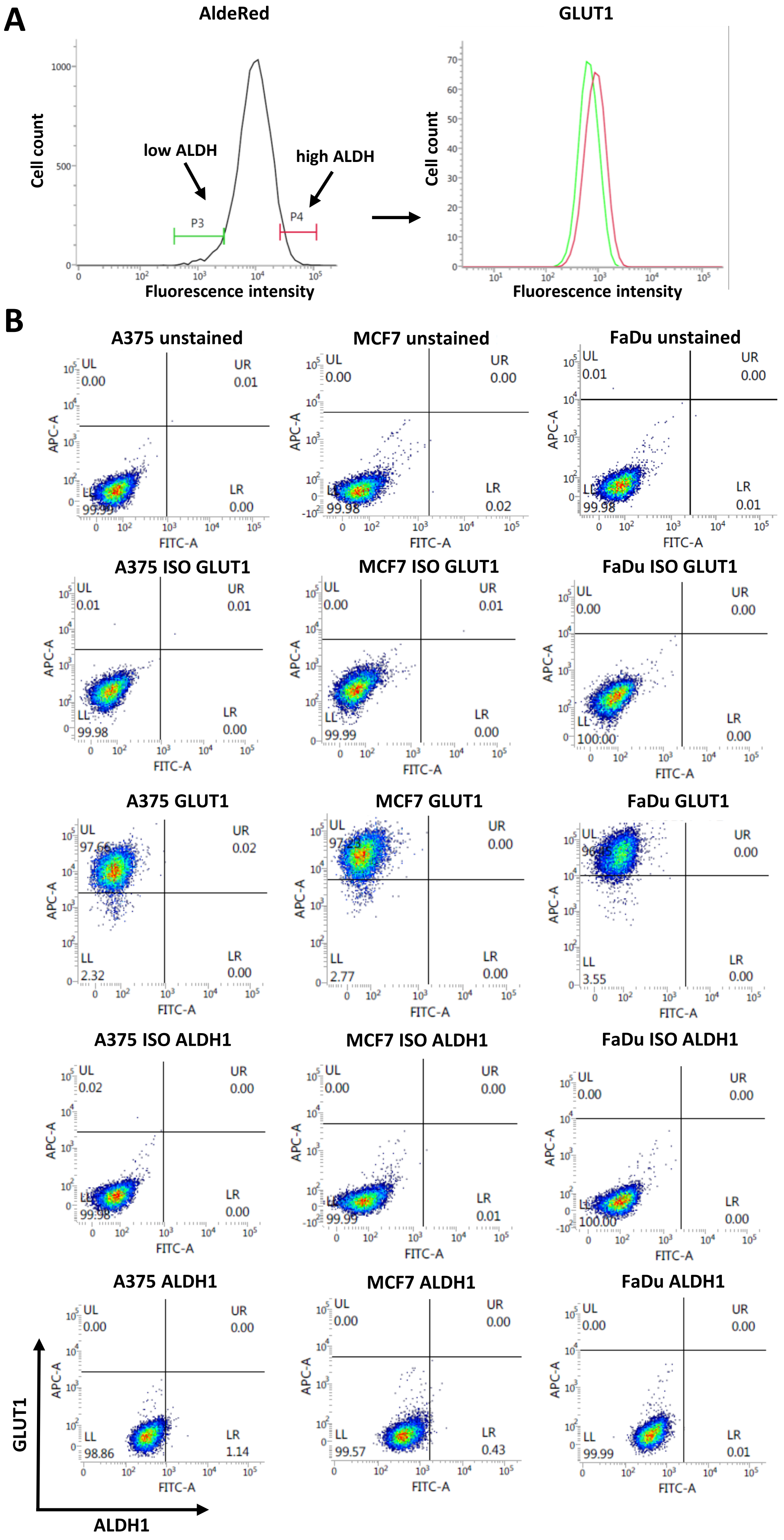

Suppl. Figure 3: ALDH activity vs glucose transport in A375, MCF7 and FaDu cells
(A) Representative histogram with fluorescence shift of GLUT1 according to the AldeRed in parental A375 population. **(B)** Glucose transport (GLUT1) and ALDH1 activity in A375, MCF7 and FaDu cells. Density plots are representatives from three independent experiments. ISO refers to Isotype control for AF647-conjugated GLUT1 antibody or AF488-conjugated ALDH1 antibody.

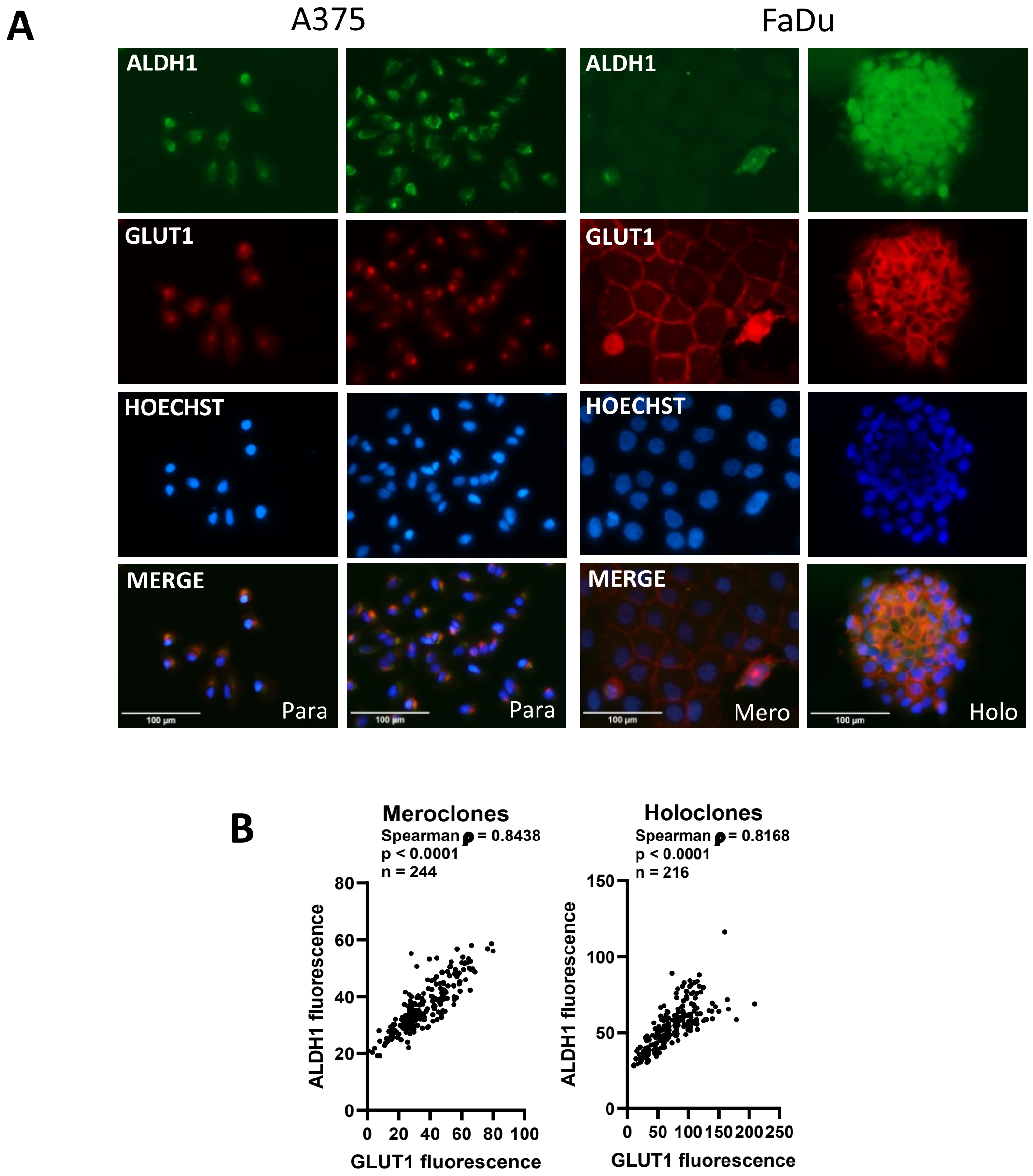

Suppl. Figure 4: ALDH activity vs glucose transport in A375, MCF7 and FaDu cells

(A) Representative immunofluorescence pictures of A375 and FaDu cells grown in low density colonies stained with ALDH1 (green), GLUT1 (red) and Hoechst (blue). The scale bar represents 100 µm. Holo/Mero/Para clone next to the scale bar denote the colony morphology type. **(B)** Fluorescence quantification of FaDu holoclones and meroclones using QuPath. Pairwise associations between markers were assessed using Spearman's rank correlation. Correlation coefficients are reported in graphs as Spearman's ρ together with associated p values. The number of analyzed cells is indicated in the graphs as n.

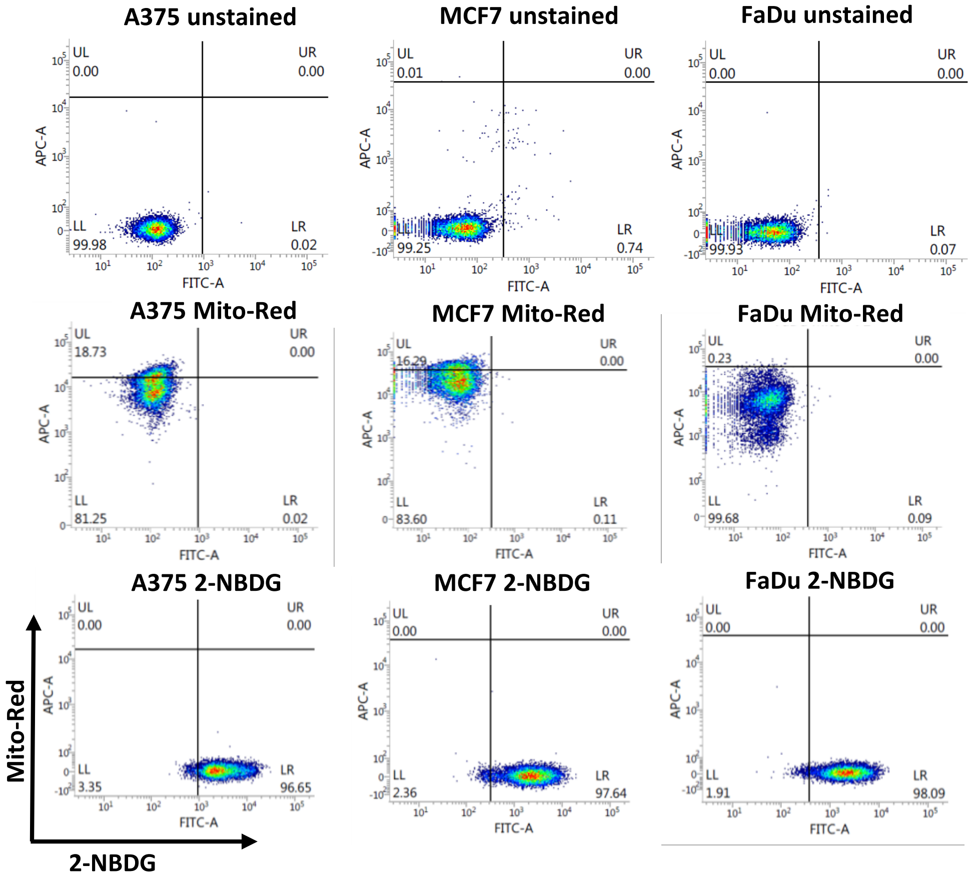

Suppl. Figure 5: Mitochondrial membrane potential vs glucose transport

Flow cytometry of mitochondrial membrane potential (Mito-Red) and glucose uptake (2-NBDG) in A375, MCF7, and FaDu cells. Density plots are representatives from three independent experiments.

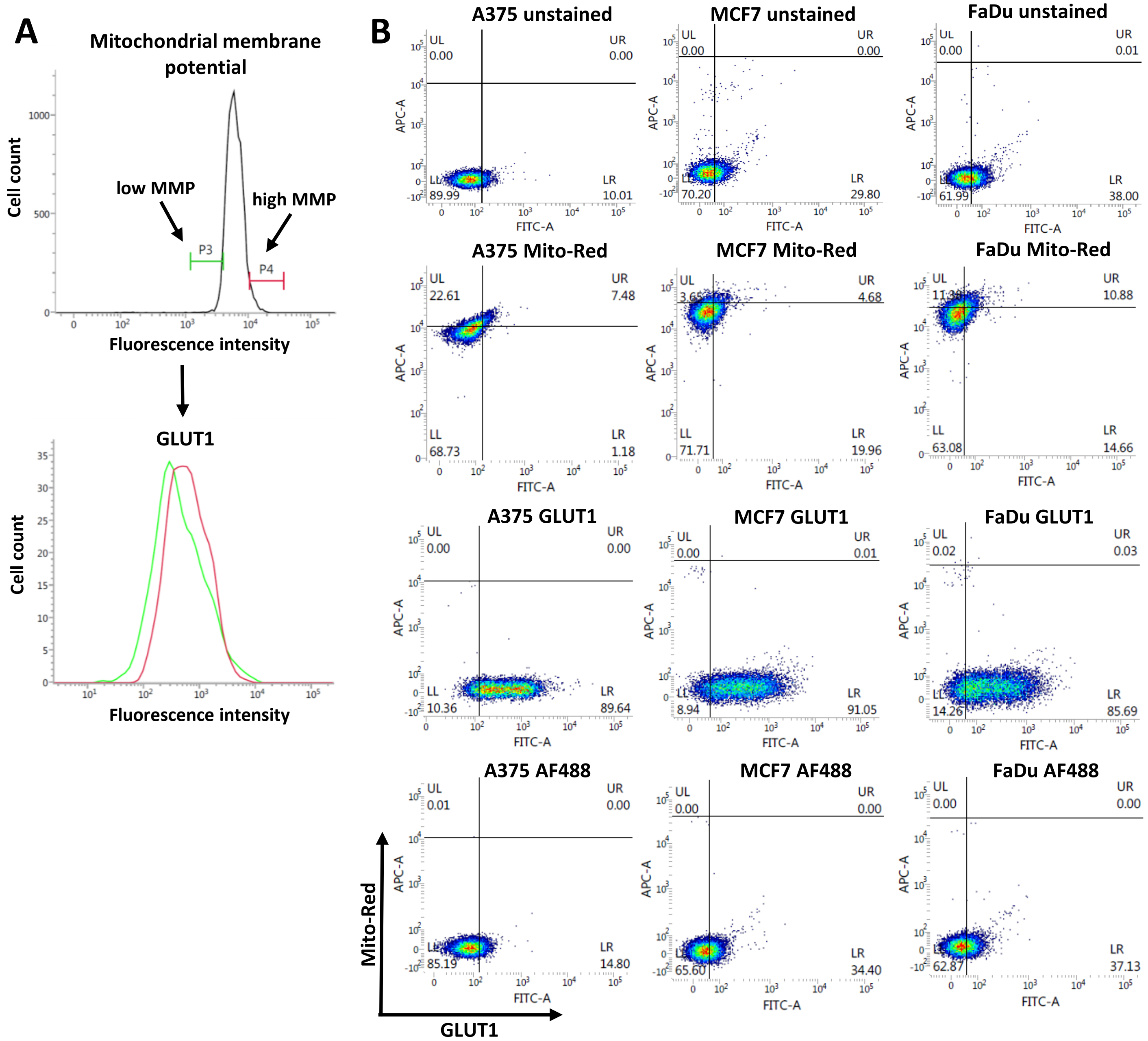

Suppl. Figure 6: Mitochondrial membrane potential vs glucose transport

(A) Histogram with fluorescence shift of GLUT1 according to the Mito-Red in parental A375 population.

(B) Flow cytometry of mitochondrial membrane potential and glucose transport in A375, MCF7, and FaDu cells. Density plots are representatives from three independent experiments.

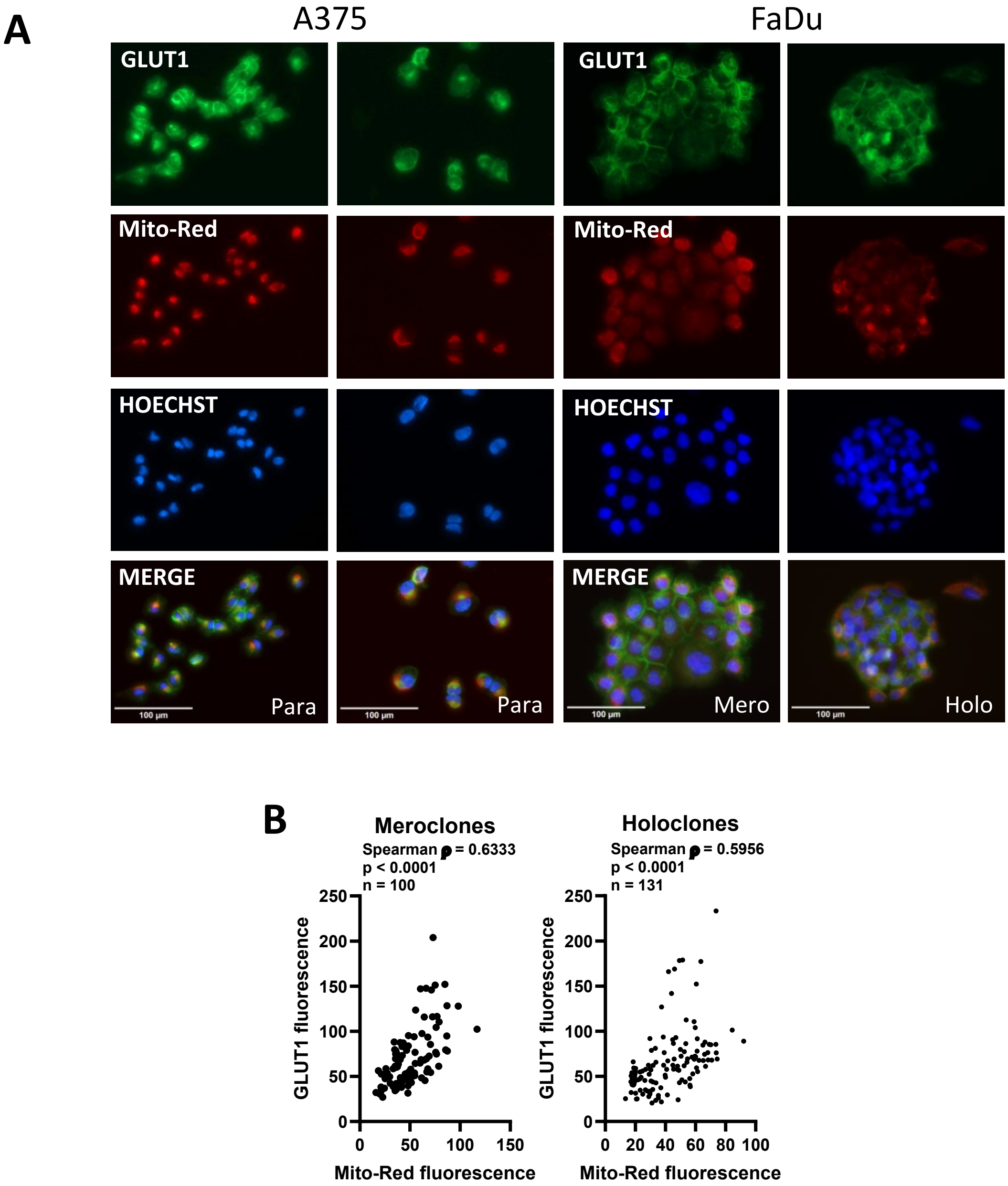

Suppl. Figure 7: Mitochondrial membrane potential vs glucose transport in A375, MCF7 and FaDu cells (A) Representative immunofluorescence pictures of A375 and FaDu cells grown in low density colonies stained with GLUT1 (green), Mito-Red (red) and Hoechst (blue). The scale bar represents 100 µm. Holo/Mero/Para clone next to the scale bar denote the colony morphology type. **(B)** Fluorescence quantification of FaDu holoclones and meroclones using QuPath. Pairwise associations between markers were assessed using Spearman's rank correlation. Correlation coefficients are reported in graphs as Spearman's ρ together with associated p values. The number of analyzed cells is indicated in the graphs as n.

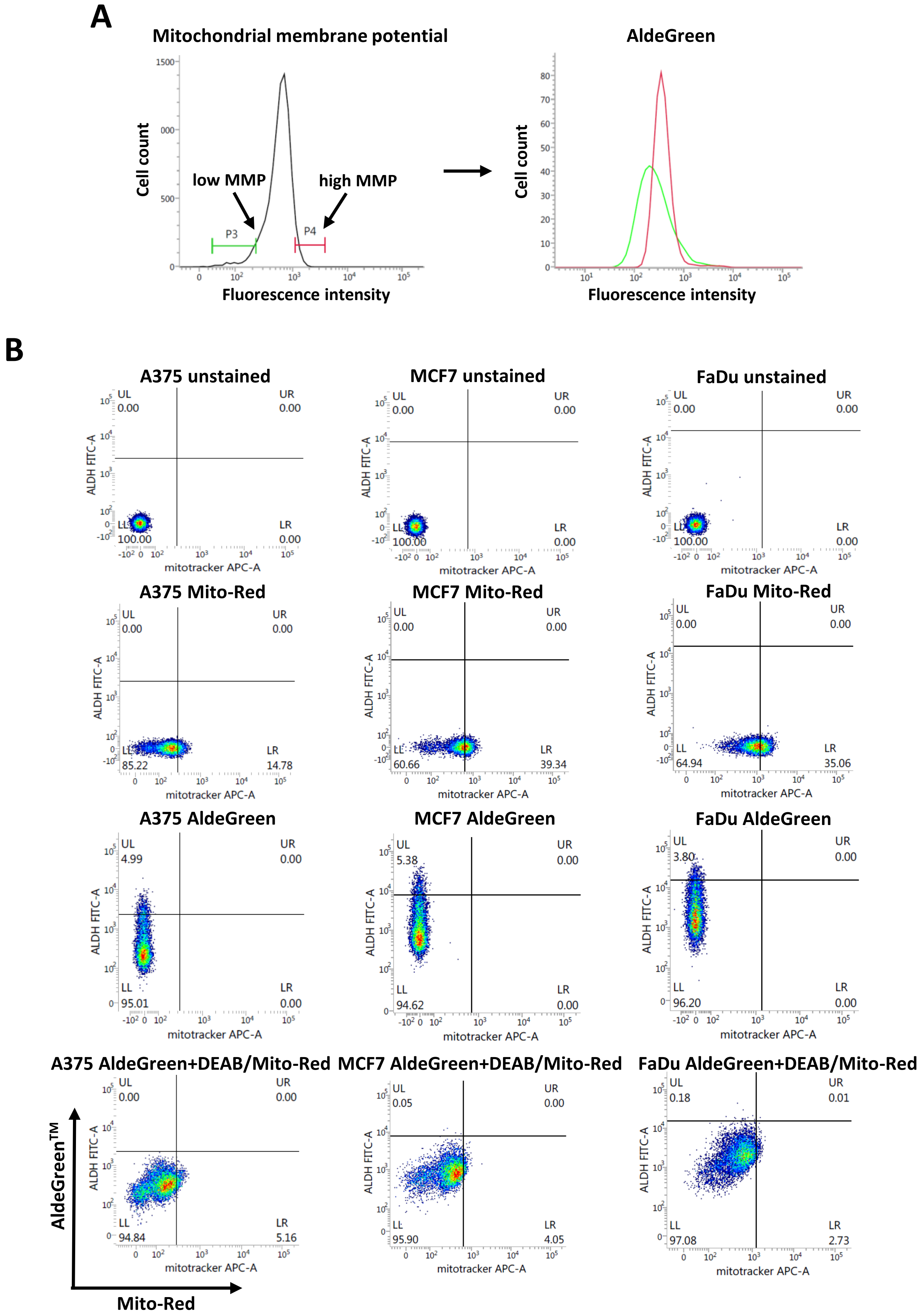

Suppl. Figure 8: ALDH activity vs mitochondrial membrane potential

(A) Representative histogram with fluorescence shift of ALDH (AldeRed) according to the mitochondrial membrane potential (Mito-Red) in parental A375 population. **(B)** Flow cytometry detection of ALDH activity and mitochondrial membrane potential in A375, MCF7 and FaDu cells. Density plots are representatives from three independent experiments.

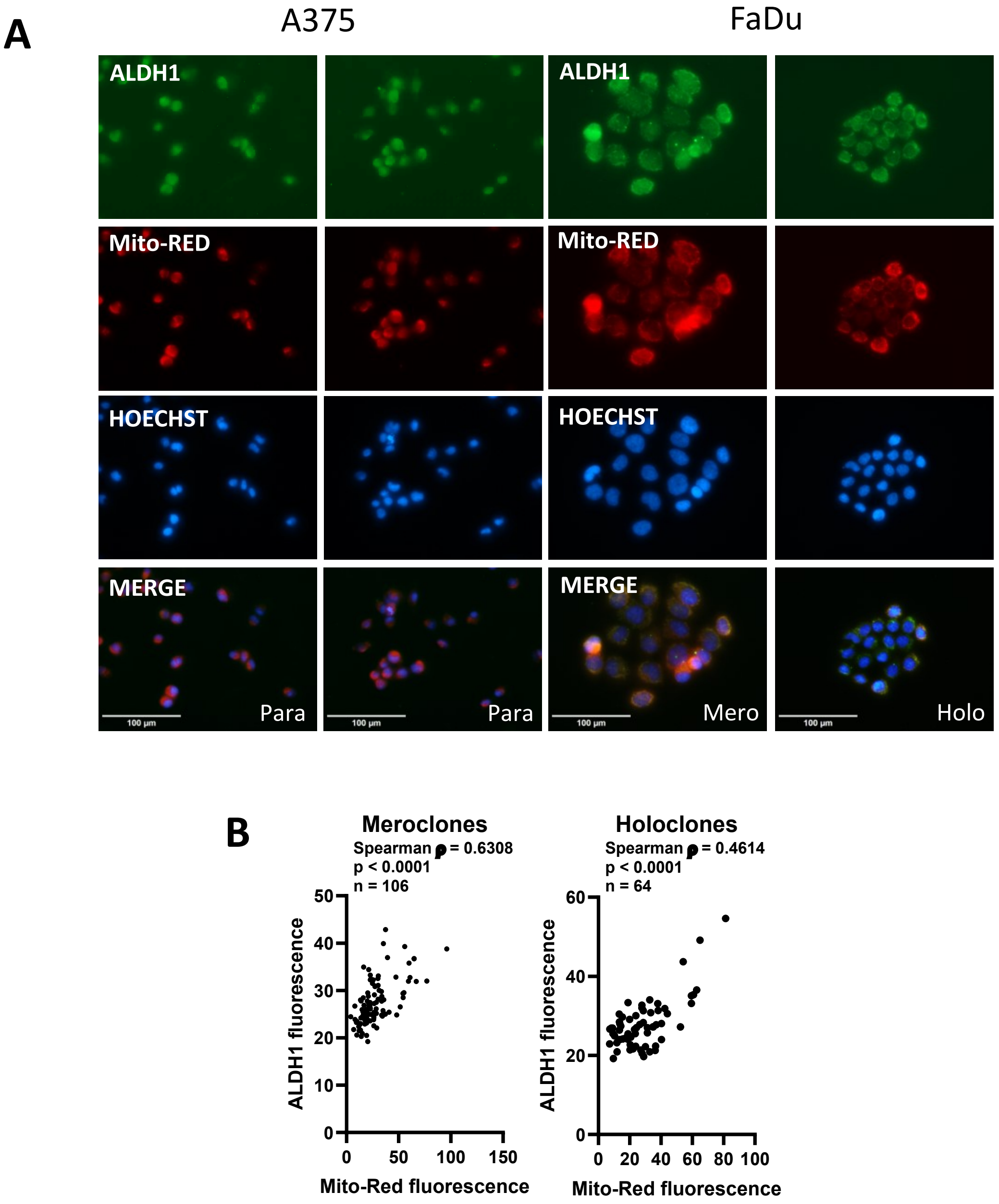

Suppl. Figure 9: ALDH activity vs mitochondrial membrane potential

(A) Representative immunofluorescence pictures of A375 and FaDu cells grown in low density colonies stained with ALDH1 (green), Mito-Red (red) and Hoechst (blue). The scale bar represents 100 µm. Holo/Mero/Para clone next to the scale bar denote the colony morphology type. **(B)** Fluorescence quantification of FaDu holoclones and meroclones using QuPath. Pairwise associations between markers were assessed using Spearman's rank correlation. Correlation coefficients are reported in graphs as Spearman's ρ together with associated p values. The number of analyzed cells is indicated in the graphs as n.

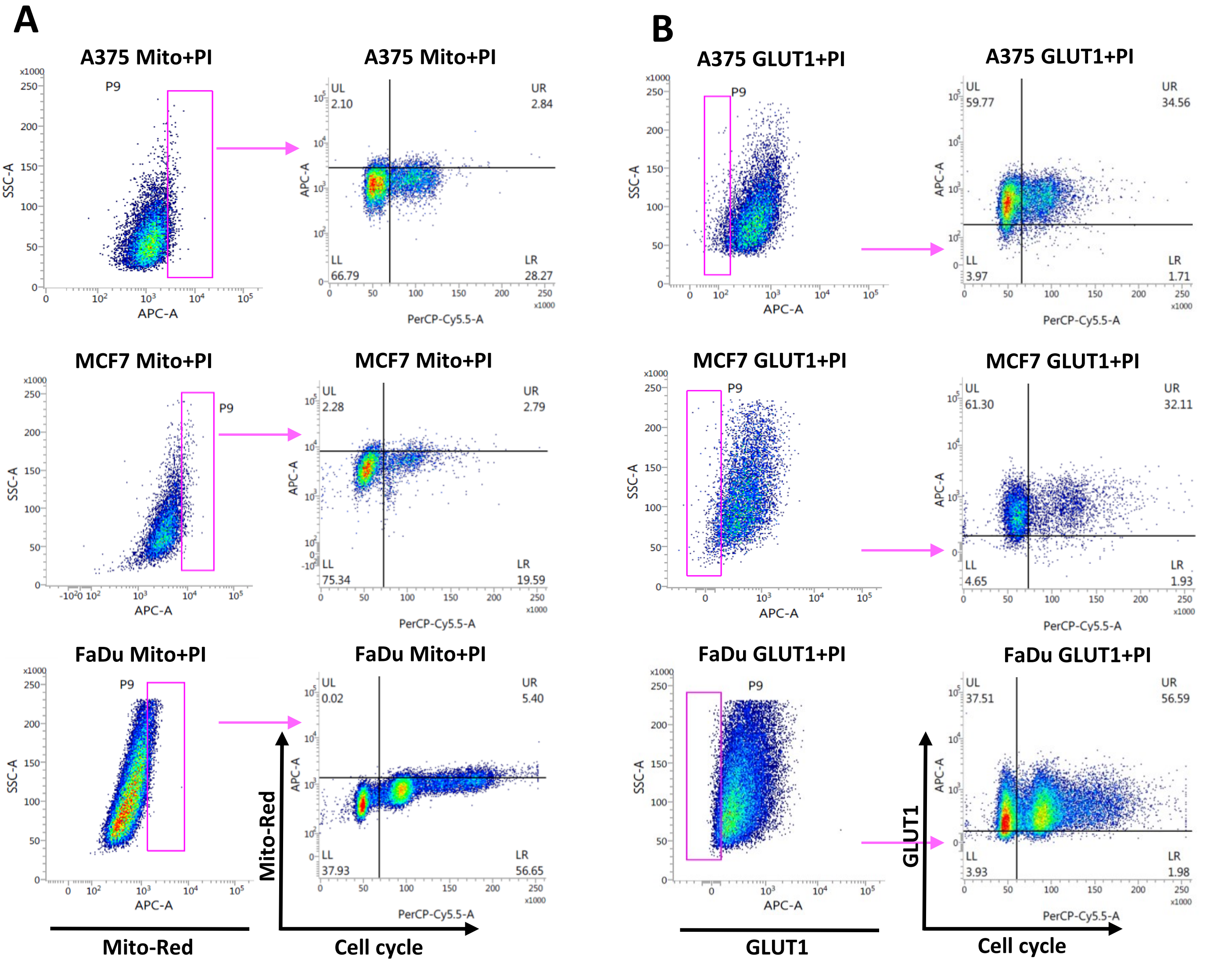

Suppl. Figure 10: Cell cycle distribution in cells with high mitochondrial membrane potential or low GLUT1 in A375, MCF7 and FaDu cells

Flow cytometry detection of **(A)** mitochondrial membrane potential and **(B)** glucose transport (GLUT1) in combination with cell cycle in control untreated A375, MCF7 and FaDu cells. Pink arrows indicate cell cycle distribution of cells with high mitochondrial membrane potential (A, top 5%, quadrants UL+UR) or low GLUT1 (B, lowest 5%, quadrants LL+LR). Density plots are representatives from three independent experiments.

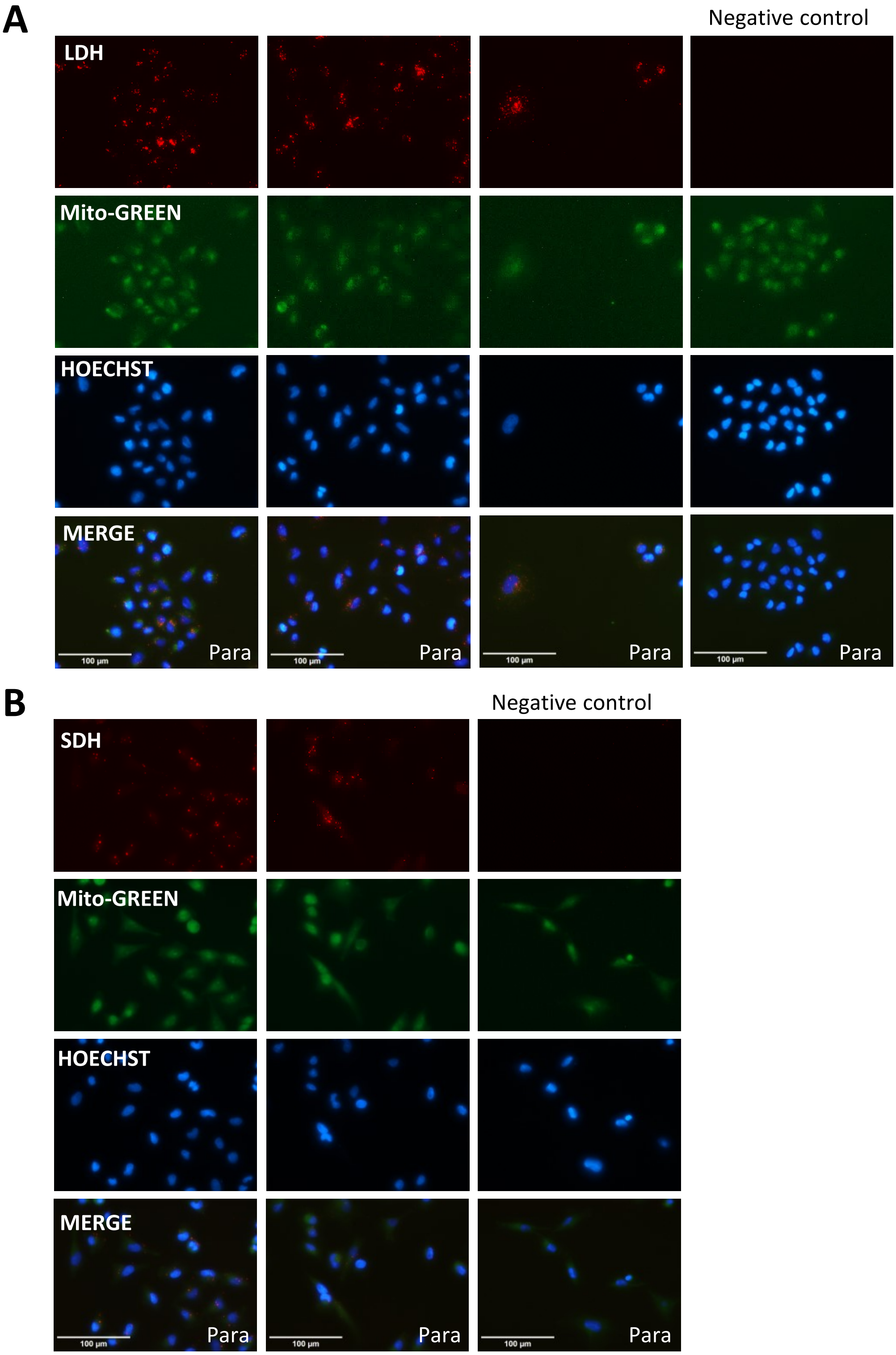

Suppl. Figure 11: LDH and SDH activity in A375 cells

Representative pictures of **(A)** LDH and **(B)** SDH activity (red) and colocalization with mitochondria (green) in A375 cells grown in low density colonies. Negative controls were prepared without substrate. The scale bar represents 100 µm. Holo/Mero/Para clone next to the scale bar denote the colony morphology type.

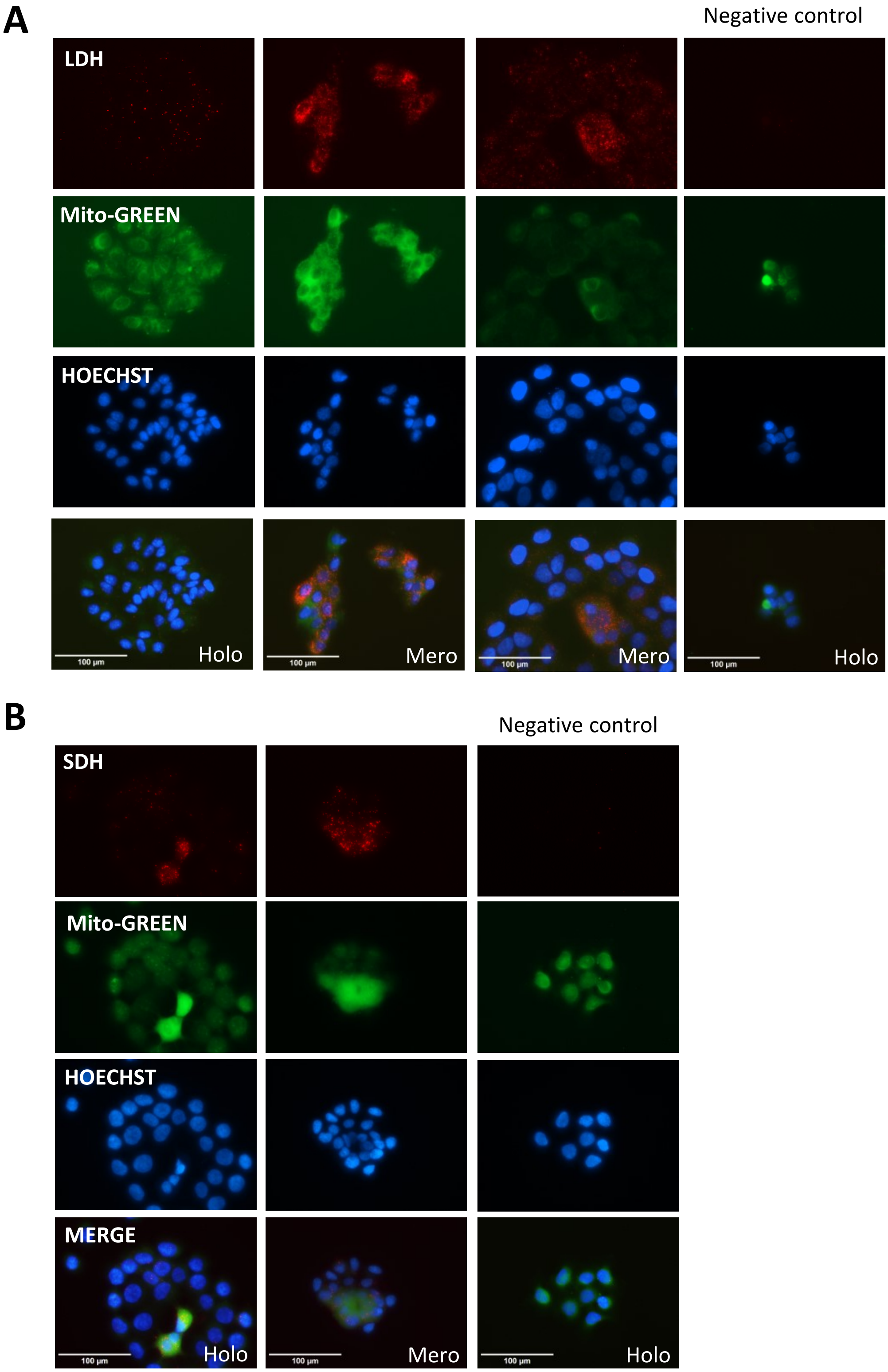

Suppl. Figure 12: LDH and SDH activity in MCF7 cells

Representative pictures of **(A)** LDH and **(B)** SDH activity (red) and colocalization with mitochondria (green) in MCF7 cells grown in low density colonies. Negative controls were prepared without substrate. The scale bar represents 100 µm. Holo/Mero/Para clone next to the scale bar denote the colony morphology type.

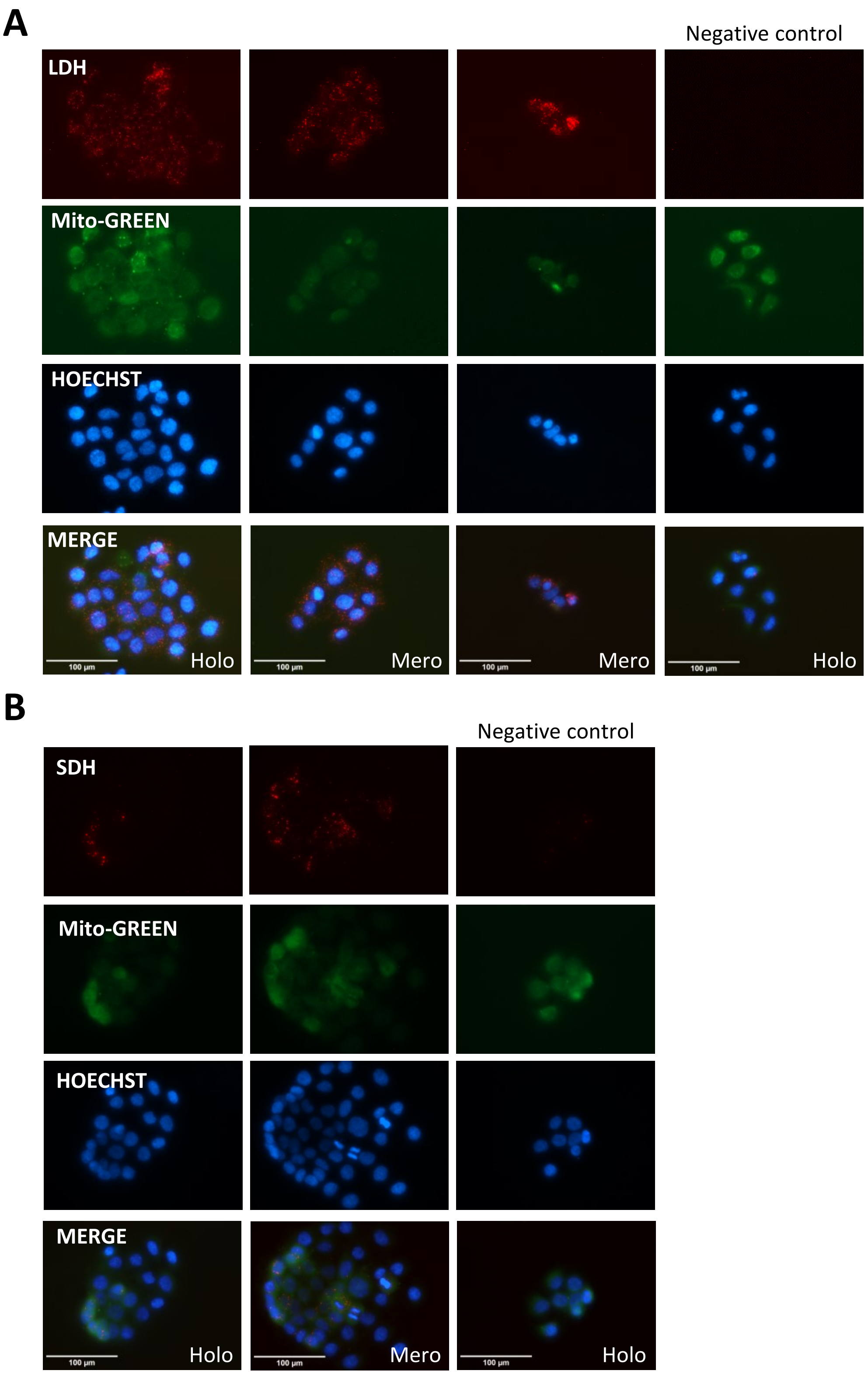

Suppl. Figure 13: LDH and SDH activity in FaDu cells

Representative pictures of **(A)** LDH and **(B)** SDH activity (red) and colocalization with mitochondria (green) in FaDu cells grown in low density colonies. Negative controls were prepared without substrate. The scale bar represents 100 µm. Holo/Mero/Para clone next to the scale bar denote the colony morphology type.

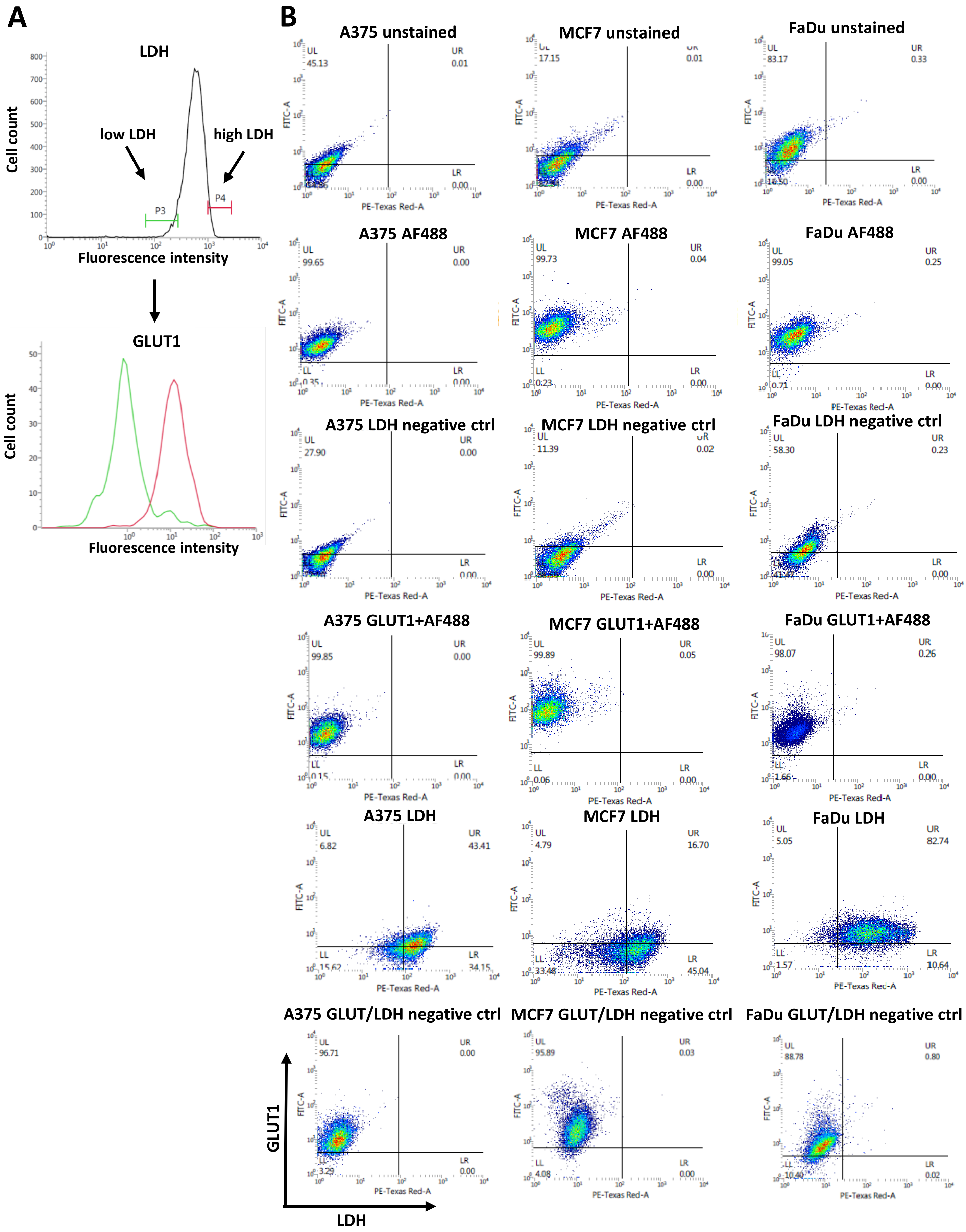

Suppl. Figure 14: LDH activity vs glucose transport

(A) Representative histogram with fluorescence shift of GLUT1 according to the LDH activity in parental A375 population. **(B)** Flow cytometry detection of LDH and glucose transport (GLUT1) in A375, MCF7 and FaDu cells. Density plots are representatives from three independent experiments.

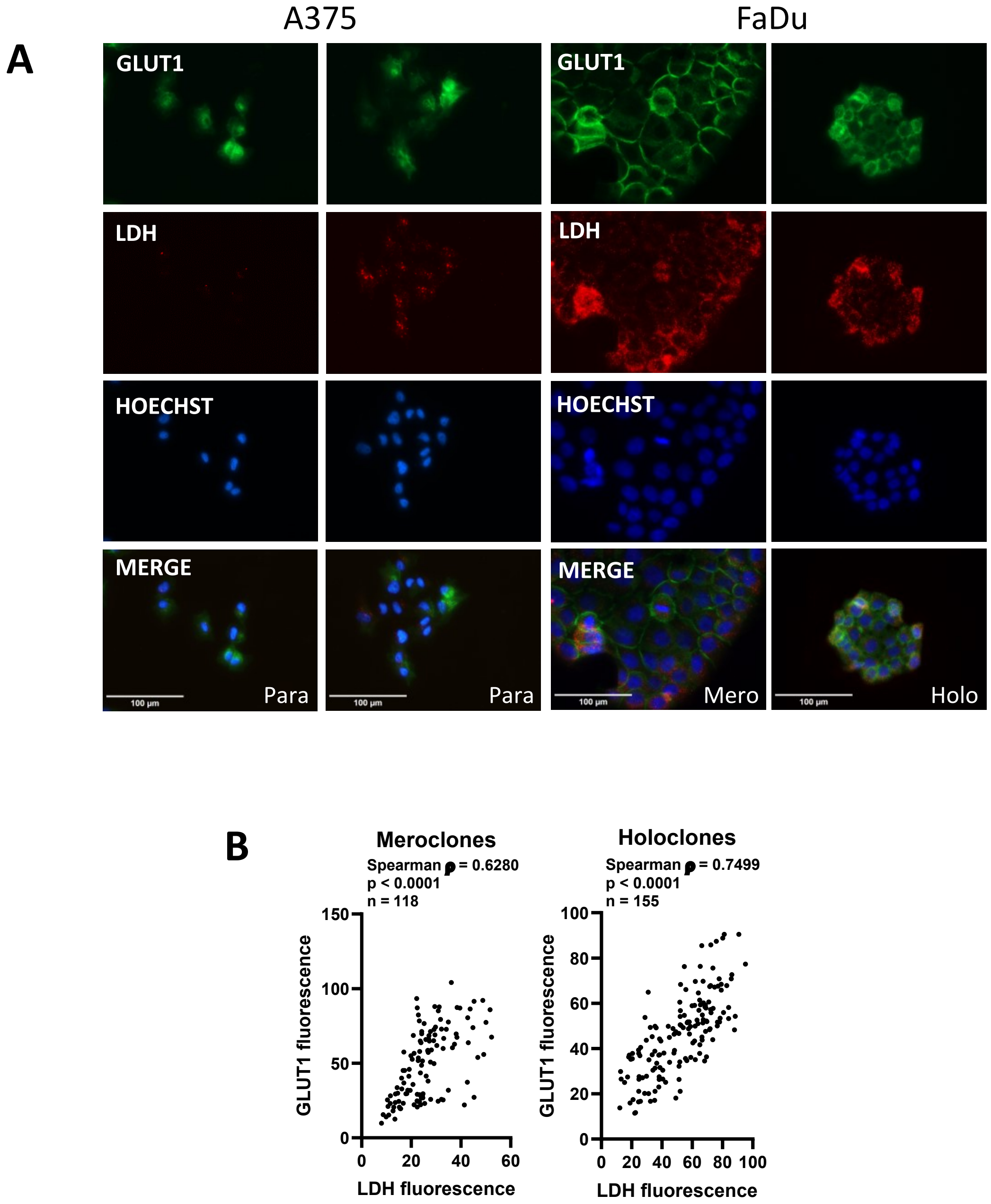

Suppl. Figure 15: LDH activity vs glucose transport

(A) Representative immunofluorescence pictures of A375 and FaDu cells grown in low density colonies stained with GLUT1 (green), LDH (red) and Hoechst (blue). The scale bar represents 100 µm. Holo/Mero/Para clone next to the scale bar denote the colony morphology type. **(B)** Fluorescence quantification of FaDu holoclones and meroclones using QuPath. Pairwise associations between markers were assessed using Spearman's rank correlation. Correlation coefficients are reported in graphs as Spearman's ρ together with associated p values. The number of analyzed cells is indicated in the graphs as n.

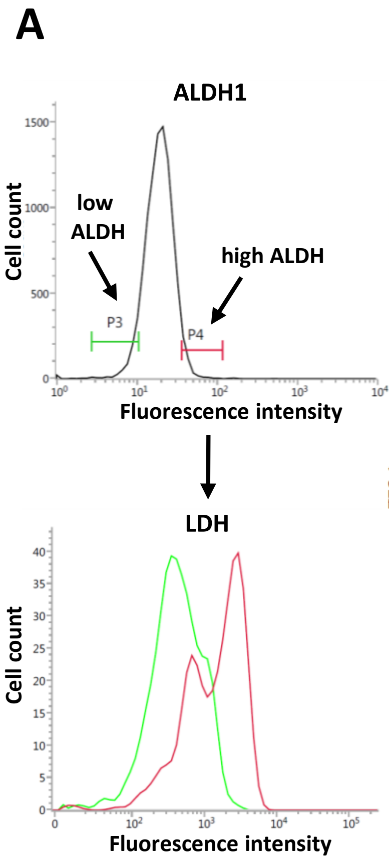

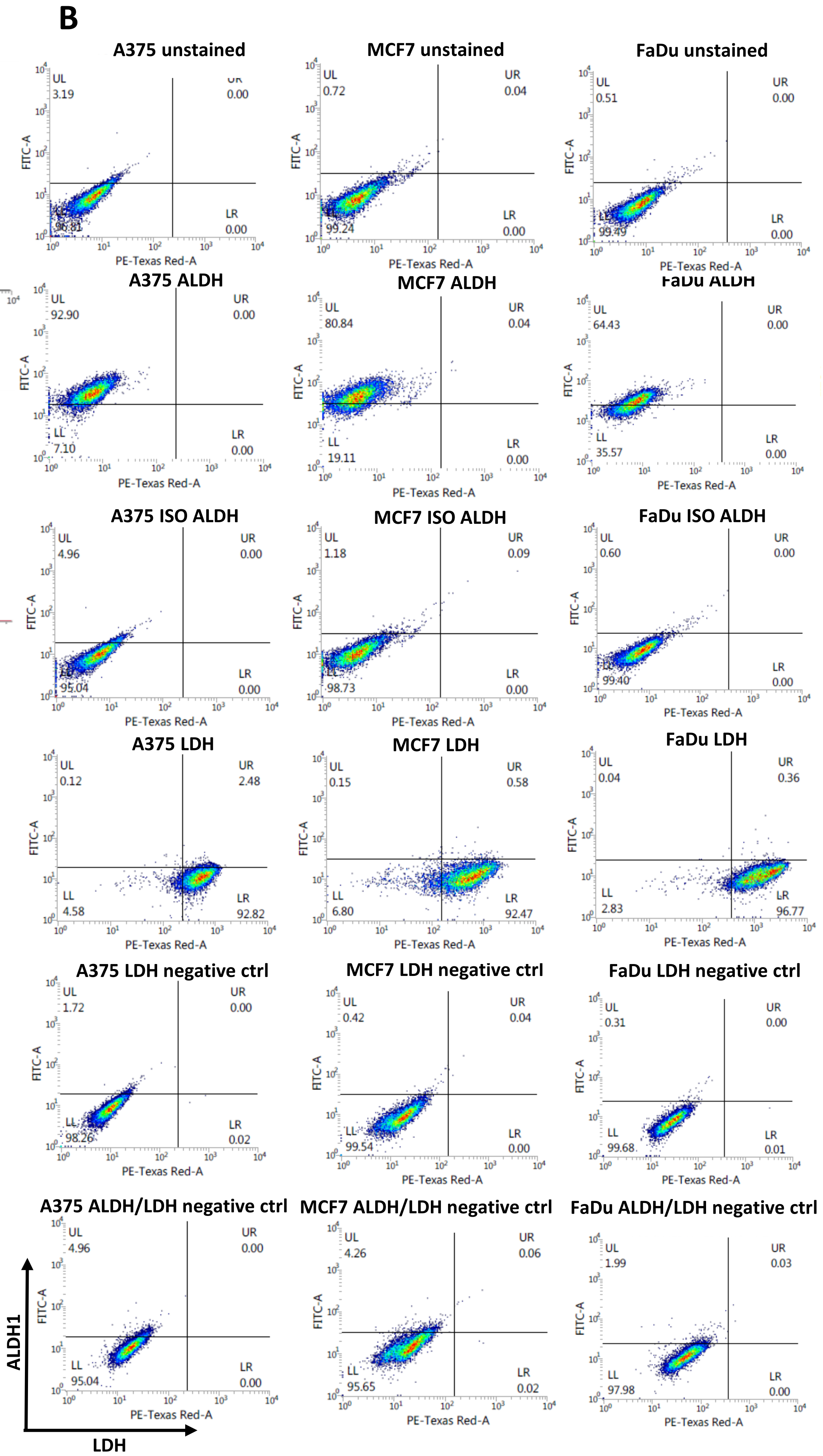

Suppl. Figure 16: ALDH activity vs LDH activity

(A) Histogram with fluorescence shift of LDH according to the ALDH1 activity in parental A375 population. **(B)** Flow cytometry detection of ALDH1 and LDH in A375, MCF7 and FaDu cells. Density plots are representatives from three independent experiments. ISO refers to Isotype control for AF488-conjugated ALDH1 antibody.

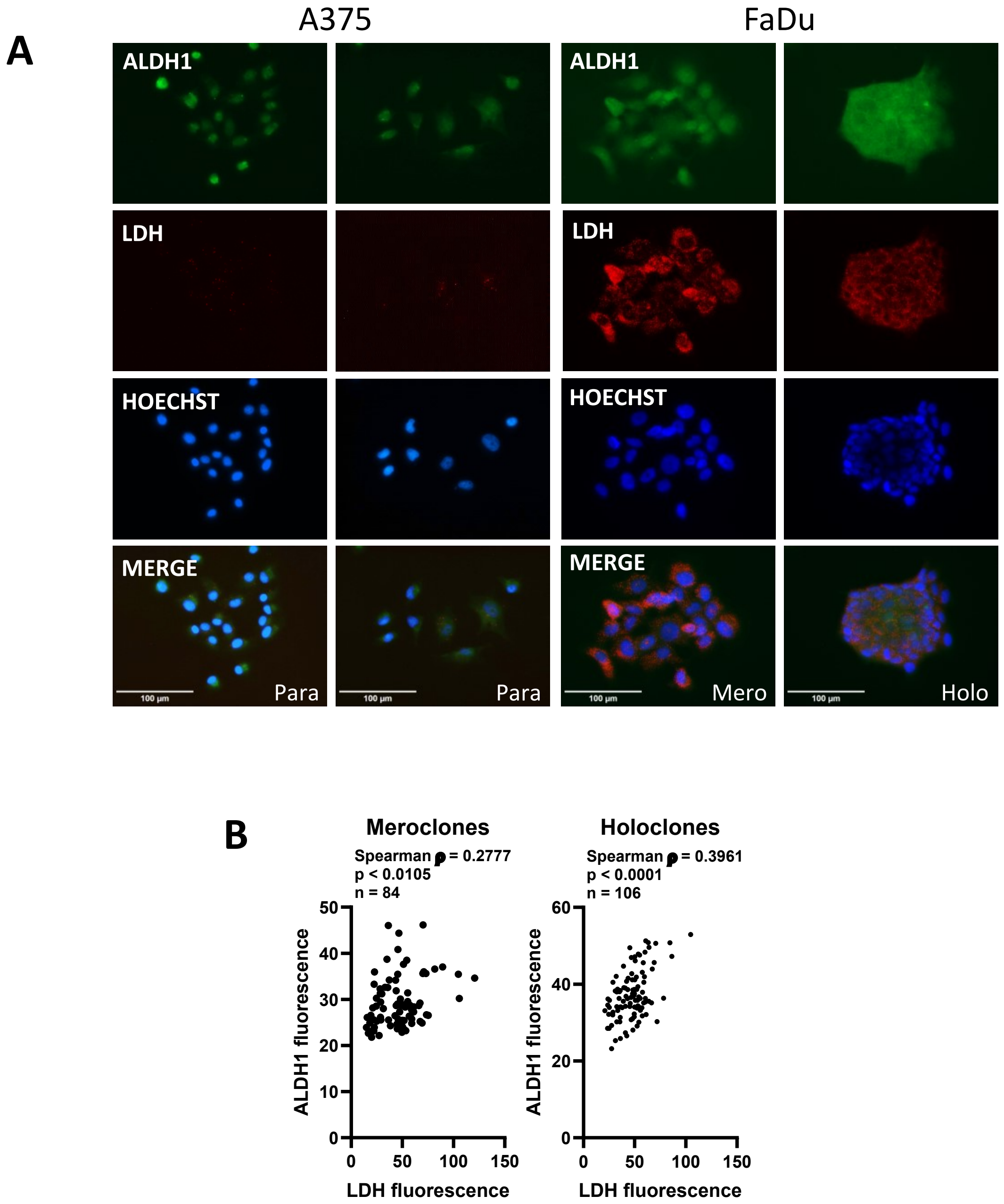

Suppl. Figure 17: ALDH activity vs LDH activity

Representative immunofluorescence pictures of A375 and FaDu cells grown in low density colonies stained with ALDH1 (green), LDH (red) and Hoechst (blue). The scale bar represents 100 µm. Holo/Mero/Para clone next to the scale bar denote the colony morphology type. **(B)** Fluorescence quantification of FaDu holoclones and meroclones using QuPath. Pairwise associations between markers were assessed using Spearman's rank correlation. Correlation coefficients are reported in graphs as Spearman's ρ together with associated p values. The number of analyzed cells is indicated in the graphs as n.

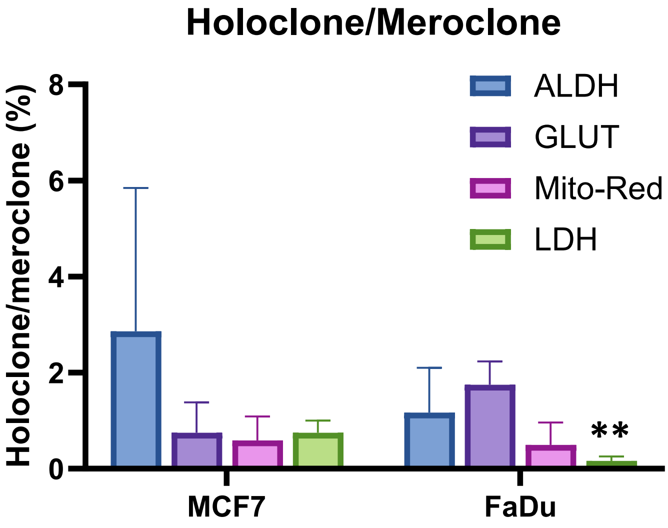

Suppl. Figure 18: Analysis on the numbers of strongly fluorescent cells in holoclones and meroclones
The mean ratio of cells with the top 5% fluorescence intensity for the indicated parameter in holoclones compared to meroclones in MCF7 and FaDu cells. Error bars represent SD, ** $p < 0.01$; n=9 for ALDH1 and GLUT1, n=6 for Mito-Red and LDH.

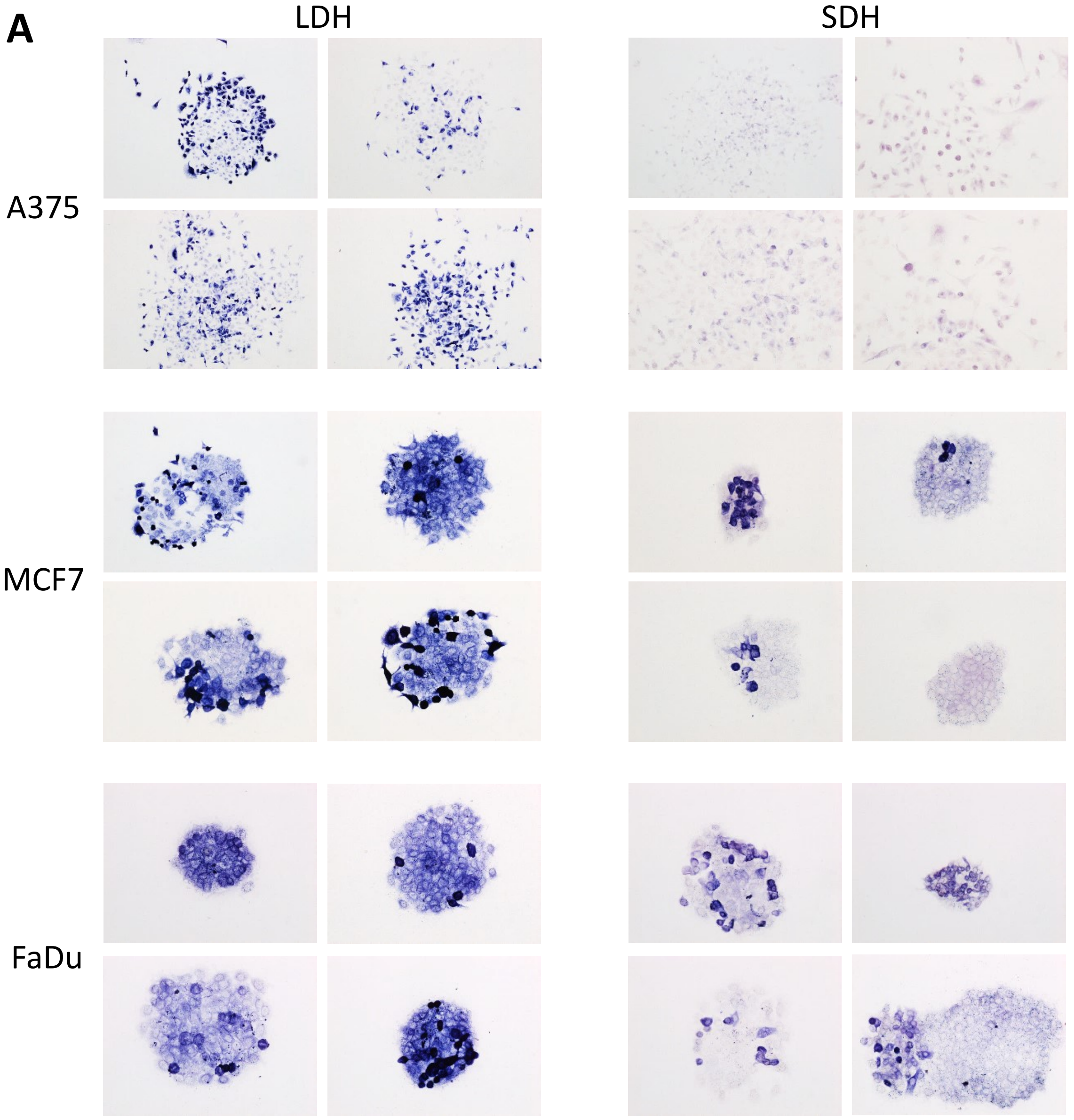

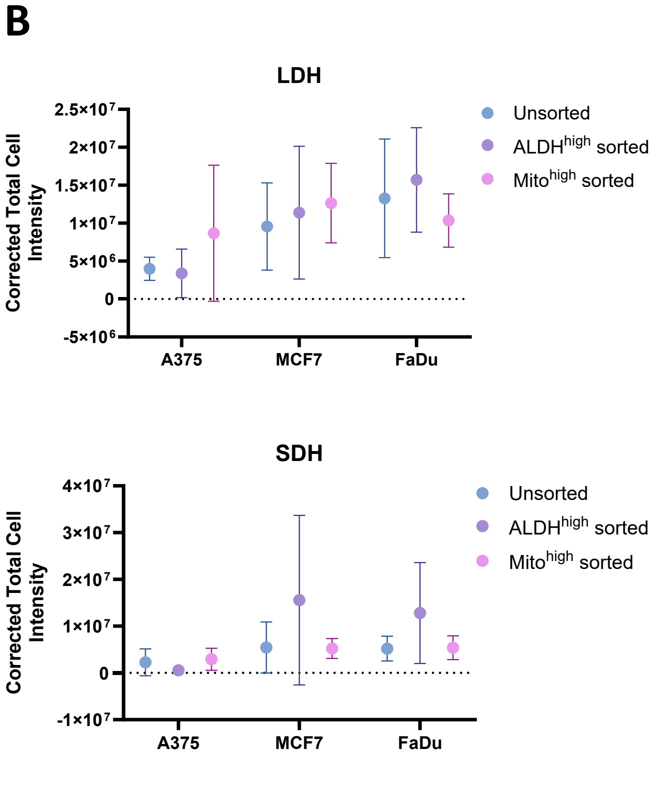

Suppl. Figure 19: LDH and SDH activity in Mito-Red^{high} sorted cells

(A) Representative pictures of LDH and SDH activity in A375, MCF7 and FaDu cells grown in low density colonies after sorting the 5% cells with highest mitochondrial membrane potential. Enzyme histochemistry was made with NBT as detection reagent. Negative controls were prepared without substrate (data not shown). **(B)** Measured intensity of sorted ALDH^{high} and Mito^{high} cells compared to control unsorted cells. 5 representative colonies per group were quantified using ImageJ, values were background-corrected by subtracting mean intensity of a reference ROI and expressed as relative NBT signal. Data are shown as mean ± SEM. Differences among unsorted, ALDH^{high}-sorted and Mito^{high}-sorted colonies were tested separately for each cell line and enzyme using one-way ANOVA and Kruskal–Wallis test, followed by post-hoc comparisons versus the unsorted control (Dunn's test with Holm correction). No statistically significant differences were detected.
